# A neural network model to screen feature genes for pancreatic cancer

**DOI:** 10.1186/s12859-023-05322-z

**Published:** 2023-05-11

**Authors:** Jing Huang, Yuting Zhou, Haoran Zhang, Yiming Wu

**Affiliations:** 1 Department of Gastroenterology, First Hospital of Jiaxing, Jiaxing, 314001 Zhejiang China; 2 Department of Respiratory, The 904Th Hospital of Joint Logistic Support Force of PLA, Affiliated Hospital of Jiangnan University, Wuxi, 214000 Jiangsu China

**Keywords:** Pancreatic cancer, Neural network model, Biomarkers, Gene expression profiling, Random forest

## Abstract

All the time, pancreatic cancer is a problem worldwide because of its high degree of malignancy and increased mortality. Neural network model analysis is an efficient and accurate machine learning method that can quickly and accurately predict disease feature genes. The aim of our research was to build a neural network model that would help screen out feature genes for pancreatic cancer diagnosis and prediction of prognosis. Our study confirmed that the neural network model is a reliable way to predict feature genes of pancreatic cancer, and immune cells infiltrating play an essential role in the development of pancreatic cancer, especially neutrophils. ANO1, AHNAK2, and ADAM9 were eventually identified as feature genes of pancreatic cancer, helping to diagnose and predict prognosis. Neural network model analysis provides us with a new idea for finding new intervention targets for pancreatic cancer.

**Supplementary Information:**

The online version contains supplementary material available at 10.1186/s12859-023-05322-z.

## Introduction

In recent years, pancreatic cancer has shown a growing trend worldwide. The resulting death rate is also climbing. From 1990 to 2017, the number of deaths, incident cases, and disability-adjusted life-years caused by pancreatic cancer in the world has more than doubled [1], and it could become the second leading cause of death from cancer in the next 20–30 years. In the past few decades, the research and treatment of pancreatic cancer have made slow progress. The diagnosis of early pancreatic cancer is still difficult, with a 5-year survival rate of less than 10%, and most of the life expectancy after diagnosis does not exceed half a year [2]. The poor prognosis of pancreatic cancer is closely related to the difficulty of early diagnosis of the disease. The onset of pancreatic cancer is hidden, and the symptoms are abdominal pain, backache, weight loss, etc., with no specific manifestations. When the symptoms are obvious, it is difficult to perform surgical treatment, only about 20% of patients can be diagnosed and resected in time at an early stage, and the 5-year survival rate of patients undergoing surgical resection is only about 15–25% [3], the response of pancreatic cancer to most chemotherapy drugs is also poor.

Smoking, obesity, and diabetes are known as major risk factors for pancreatic cancer, and excessive alcohol intake is also one of the reasons for the increased risk of pancreatic cancer. Smoking is still the main cause of pancreatic cancer in the world, and it is currently recognized as a risk factor [4–6]. Others may be related to pancreatitis, allergy, microbial flora, and genetic susceptibility genes. At present, tri-phasic pancreatic-protocol CT is an important way to diagnose pancreatic cancer. To a great extent, it provides predictive value for resection and reference value for advanced pancreatic cancer and metastasis. However, it has poor sensitivity in detecting early pancreatic cancer. Endoscopic ultrasound is also very helpful for the diagnosis of pancreatic cancer, and diagnostic cytological sampling is performed under an endoscope. In addition, MRI also plays a certain role in the diagnosis of pancreatic cancer [7].

Artificial neural network (ANN) is a new field currently used for data analysis. It is a model built based on machine learning, which has been used to solve various tasks. Neural network take neurons as the basic unit of calculation, accept input or external data from different neurons, and then calculate an output. Different input values have a weight, and the size of the weight is used to measure the importance of the input value. ANN process information and nonlinear transformations by simulating the way the human brain processes information, not only according to a given program, but also adapt to the environment and summarize the rules, acquire external knowledge through learning and store it in the network [8]. They can process large-scale data quickly and accurately, and can accurately predict. The accuracy of the model is particularly important for studying and understanding potential molecular and cellular mechanisms [9, 10]. On many bioinformatics problems, they have shown better performance than traditional machine learning methods, such as sequence motif discovery [10, 11], chromatin interaction prediction [12], and genetic variation detection [13].

Machine learning will analyze and process a large number of data from different databases, extract the genetic information that meets our screening conditions to the greatest extent in the case of minimal human intervention, build a random forest tree. Judging its importance according to the weight of the input genetic information, perform calculations in the middle hidden layer, transform the input information of the input layer through calculation, and then output it to the output layer, conduct in-depth analysis of the output results, and clarified its attribute characteristics. After that, the accuracy of random forest tree was detected again by ROC curve. In addition, random forest tree has been widely used in predicting hospitalization rate and risk of recurrence [14].

In the gene model of cancer, the neural network model extracts cross-feature genes from multiple cancer databases and seeks genes with significant differential expression and high correlation pathways, which is an effective method to learn about the disease and further search [8]. More and more neural network models have been applied to the research of cancer, providing reference value for the early prediction and prognosis of cancer. For example, outstanding characteristics have been shown in predicting risk factors [15], or capsule network (CapsNet) in novel disease-related compound identification model-based [16]. However, there is still a lack of ideal models in pancreatic cancer, including biomarkers and pathway mechanisms, to provide complete ideas of pancreatic cancer, with exact specificity and sensitivity.

Therefore, our research aims to conduct an in-depth analysis of pancreatic cancer, by constructing a neural network model to filter out its feature genes.The data acquired from the GEO database were sorted and summarized, and the feature genes were picked out from differentially expressed genes (DEGs) as candidate biomarkers according to their importance scores. These DEGs were then validated at the transcriptome and protein levels, demonstrating the differences in their expression in pancreatic cancer tissues and their impact on prognosis.

## Methods

### Downloading of public data

Transcriptome data of three pancreatic cancer-related datasets (GSE15471, GSE16515, GSE32676) were obtained from GEO database. GSE15471 and GSE16515 were combined and used as the train group, and GSE32676 was used as the test group. In addition to this, the gene expression profiles and corresponding survival information of 178 pancreatic cancer patient samples were downloaded from the TCGA database for OS, PFS and ROC curve analysis.

### Identification of differentially expressed genes

Screening differentially expressed genes (DEGs) of train group with “limma” R package. The standard definition of the difference is logFC ≥ 1 or ≤  − 1 and adj *p* value < 0.05. The normal sample group and tumor sample group were defined as the control group and treat group respectively. Finally, the heatmap and volcano map were drawn by using the “pheatmap” and “ggplot2” R packages to show the results of differentially expressed genes.

### Enrichment analysis of DEGs

Firstly, we used Metascape (https://metascape.org/gp/index.html) to further analyze the enrichment of DEGs, including the KEGG pathway, GO Biological Processes, Reactome Gene Sets, Canonical Pathways, and so on. The group with *P* Value < 0.01, a minimum count of 3, and an enrichment factor > 1.5. Then, GO analysis and KEGG pathway analysis were performed to identify the biological function of DEGs. Finally, the protein–protein interaction (PPI) network diagram of DEGs was constructed.

### Identification of feature genes

The random forest tree was used to filter the differential genes. According to the principle of minimum cross-validation error and gene importance score > 2, the feature genes were found and depicted by the heatmap.

### Establishment of the neural network model

First of all, we scored the feature genes. According to the median value of each gene, if the gene was up-regulated, its expression in the sample will be 0 if it was less than the median value, and 1 if it was greater than the median value. Similarly, when the gene was down-regulated, the expression level in the sample was recorded as 1 when it was less than the median value, and 0 when it was greater than the median value so that the score of each gene in each sample can be obtained. Then, according to the obtained gene score, we constructed our neural network. The input layer was composed of the scores and weights of pancreatic cancer feature genes, thus obtaining the hidden layer. Then, the output layer was deduced according to the nodes and weights of the hidden layer, to judge whether the sample belongs to the control group or the treat group, thus judging the sample attributes. Then we drew the ROC curve to assess the accuracy of the neural network model in predicting sample attributes. The area under the curve is 0.5–1.0, and the closer the area is to 1, the higher the accuracy of the neural network model.

### Evaluation of immune cells infiltrating

The CiberSort algorithm was used to predict immune cell infiltration in a sample. Correlations between immune cells were tested by the Spearman coefficient and presented with correlation heatmaps. Finally, The Wilcoxon test was used to identify the distribution of immune cells in different groups and displayed as a violin diagram.

### Immunohistochemical staining images

The protein expression of feature genes in tumor and control groups was confirmed by immunohistochemistry, and images of immunohistochemistry were obtained from Human Protein Atlas (HPA). Histochemical images were divided into not detected, low, medium, and high to assess the amount of protein expressed.

### Statistical analysis

All data analysis and graphic drawings in this study were processed by R software (R version 4.2.1).

## Results

### Acquisition and grouping of raw expression profiling data

**Fig. 1:**
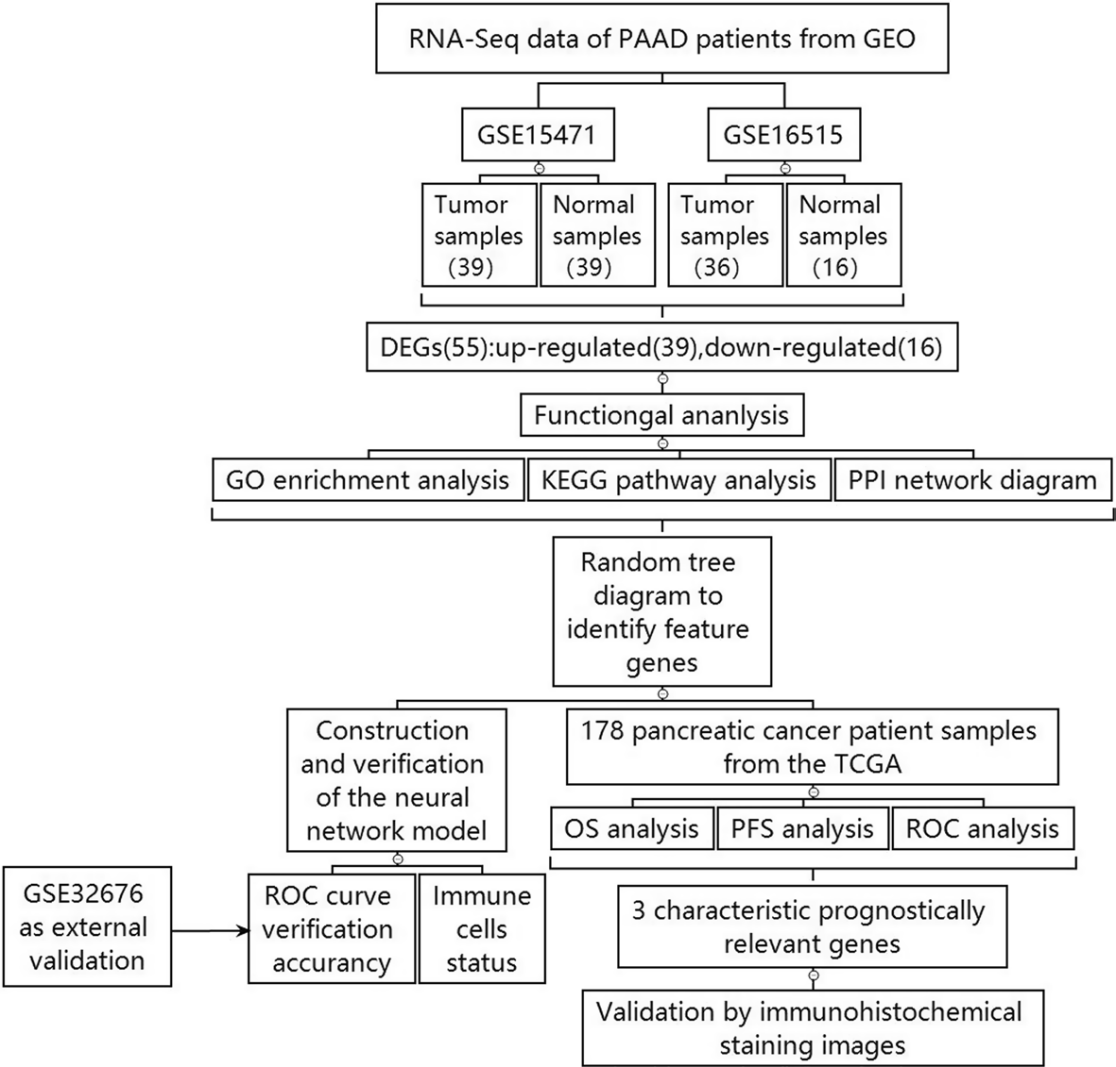
Flow diagram of the research route

The flow diagram of the study was presented in (Fig. 1). We first selected the databases with as many samples as possible according to the comparison conditions between pancreatic cancer and normal tissues from GEO databases. Finally, we chose three datasets (GSE15471, GSE16515, GSE32676), GSE15471 contains 39 normal samples and 39 tumor samples. GSE16515 contains 16 normal samples and 36 tumor samples. These two databases were combined as the train group, and GSE32676, which contains 7 normal samples and 25 tumor samples, was used as the test group.

### Identification of DEGs

**Fig. 2:**
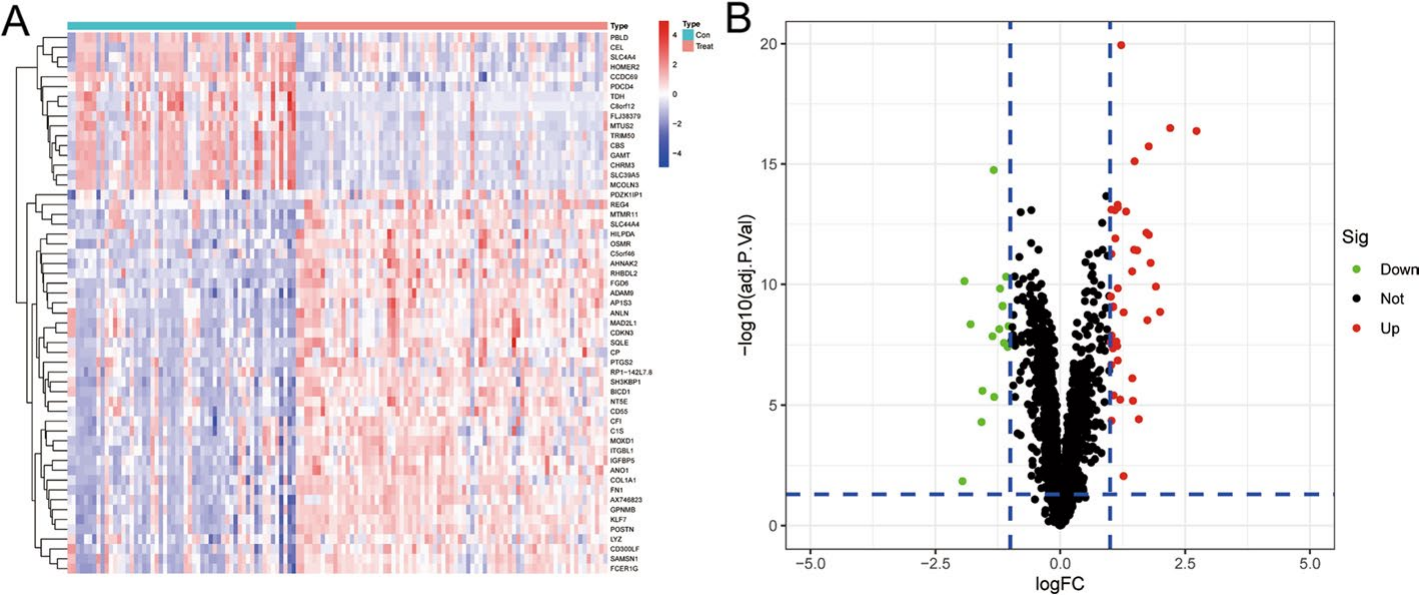
Identification of DEGs in pancreatic cancer. **A** The 39 most significantly upregulated and 16 downregulated DEGs were selected from the databases GSE15471 and GSE16515 and displayed in heatmap; **B** The DEGs were screened out under the conditions of logFC ≥ 1 or ≤  − 1 and *p* value < 0.05, and displayed in volcanic maps; DEGs, differentially expressed genes

The DEGs were screened out after comparing the train group with the test group, and according to the principle of logFC ≥ 1 or ≤  − 1 and adj. *p* value < 0.05, 55 DEGs were singled out, of which 39 were up-regulated and 16 were down-regulated in the test group. These DEGs were shown by heatmap and volcano map (Fig. 2A, B).

### Visual enrichment analysis of DEGs

**Fig. 3:**
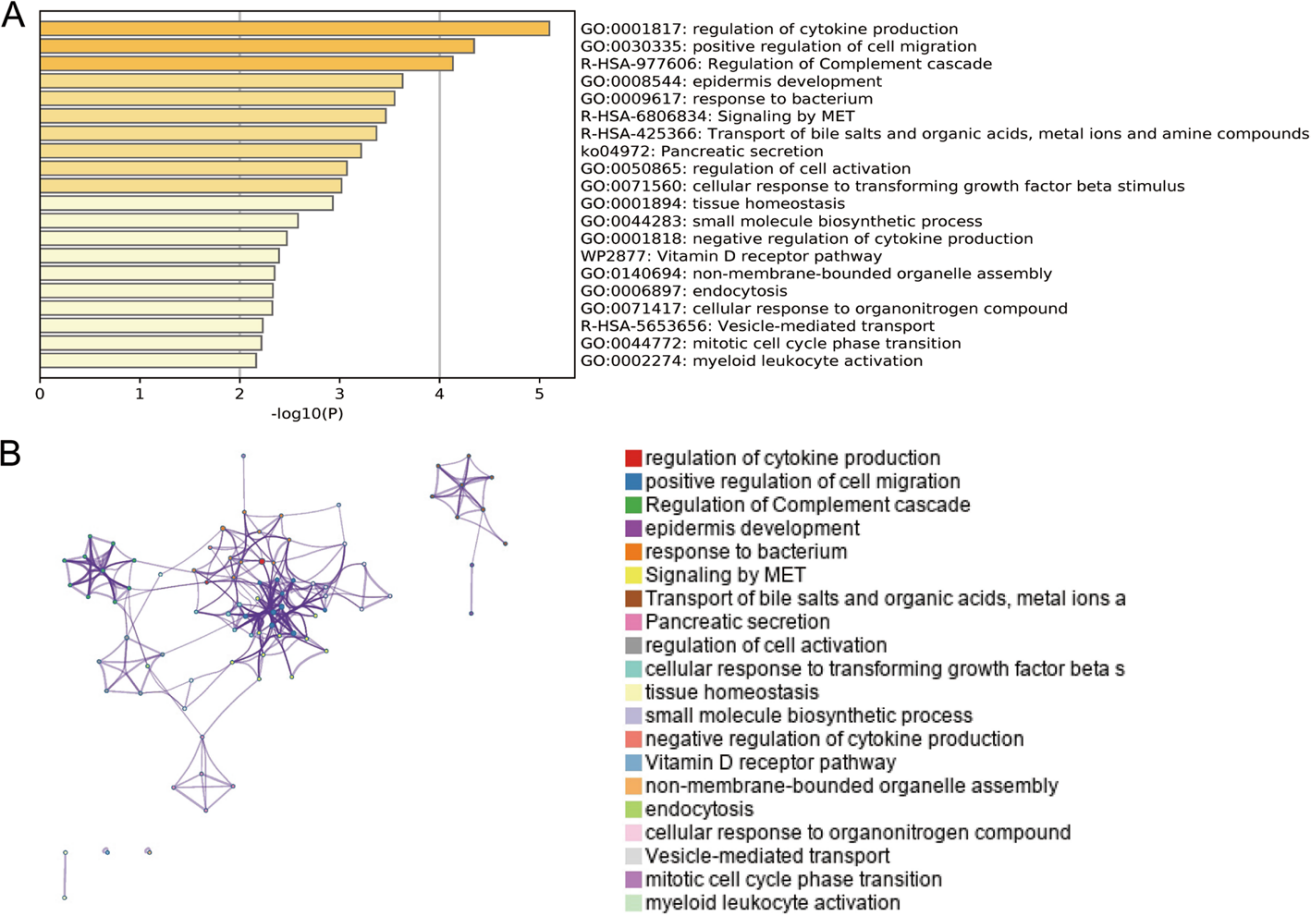
Pathway and function of DEGs enrichment. **A** Using Metascape analysis to draw bar diagram to find out the pathway and function of DEGs enrichment; **B** The network diagram showed the functions and pathways of DEGs enrichment, clustered according to node similarity, and represented in different colors

**Fig. 4:**
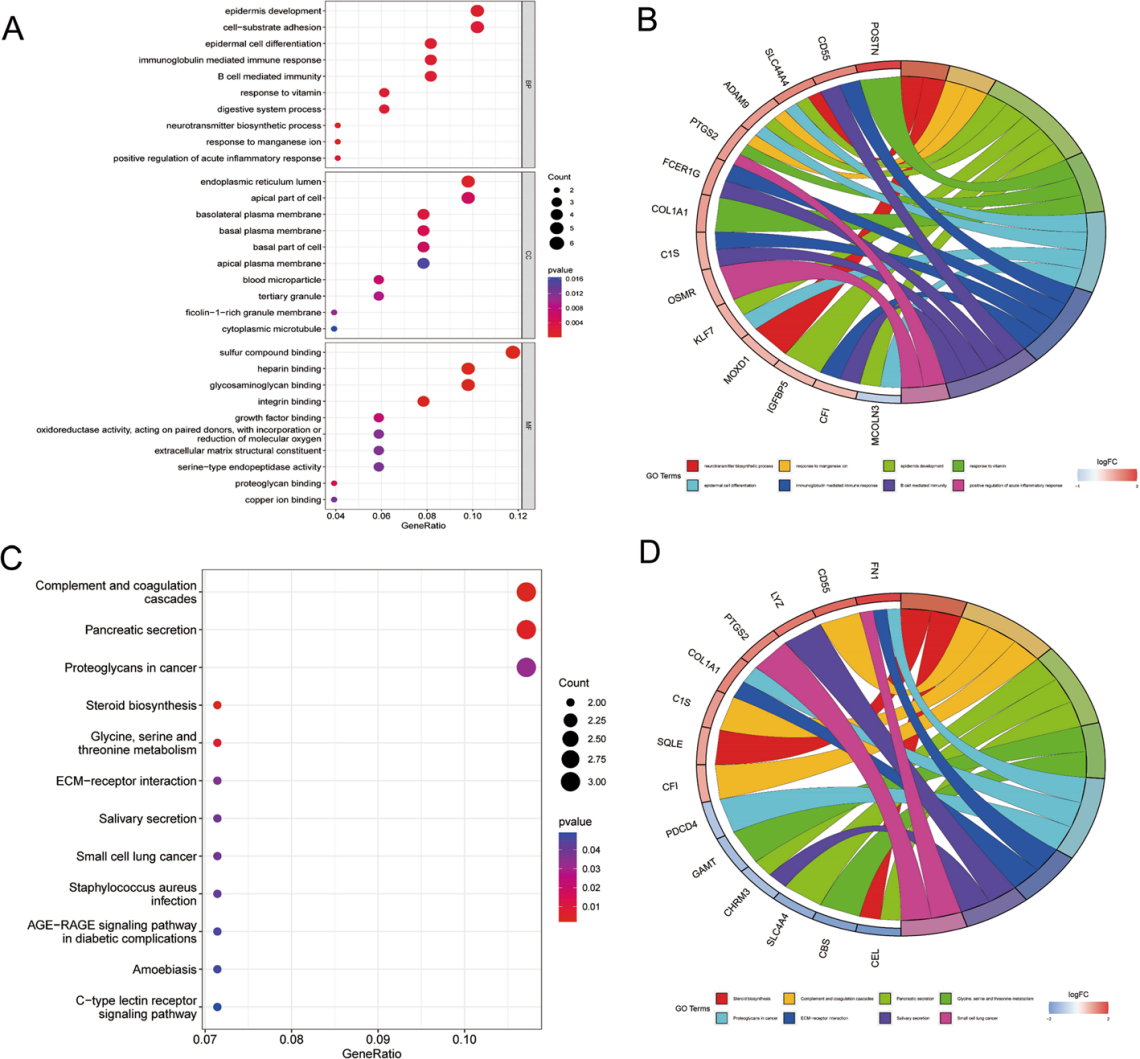
GO enrichment analysis and KEGG pathway analysis of DEGs. **A**, **B** GO enrichment analysis showed that these DEGs were mainly enriched in epidermis development(BP), endoplasmic reticulum lumen(CC), and sulfur compound binding(MF); **C**, **D** The KEGG pathway analysis revealed that the DEGs were mainly associated with Pancreatic secretion and Complement and coagulation cascades [17–19]

**Fig. 5:**
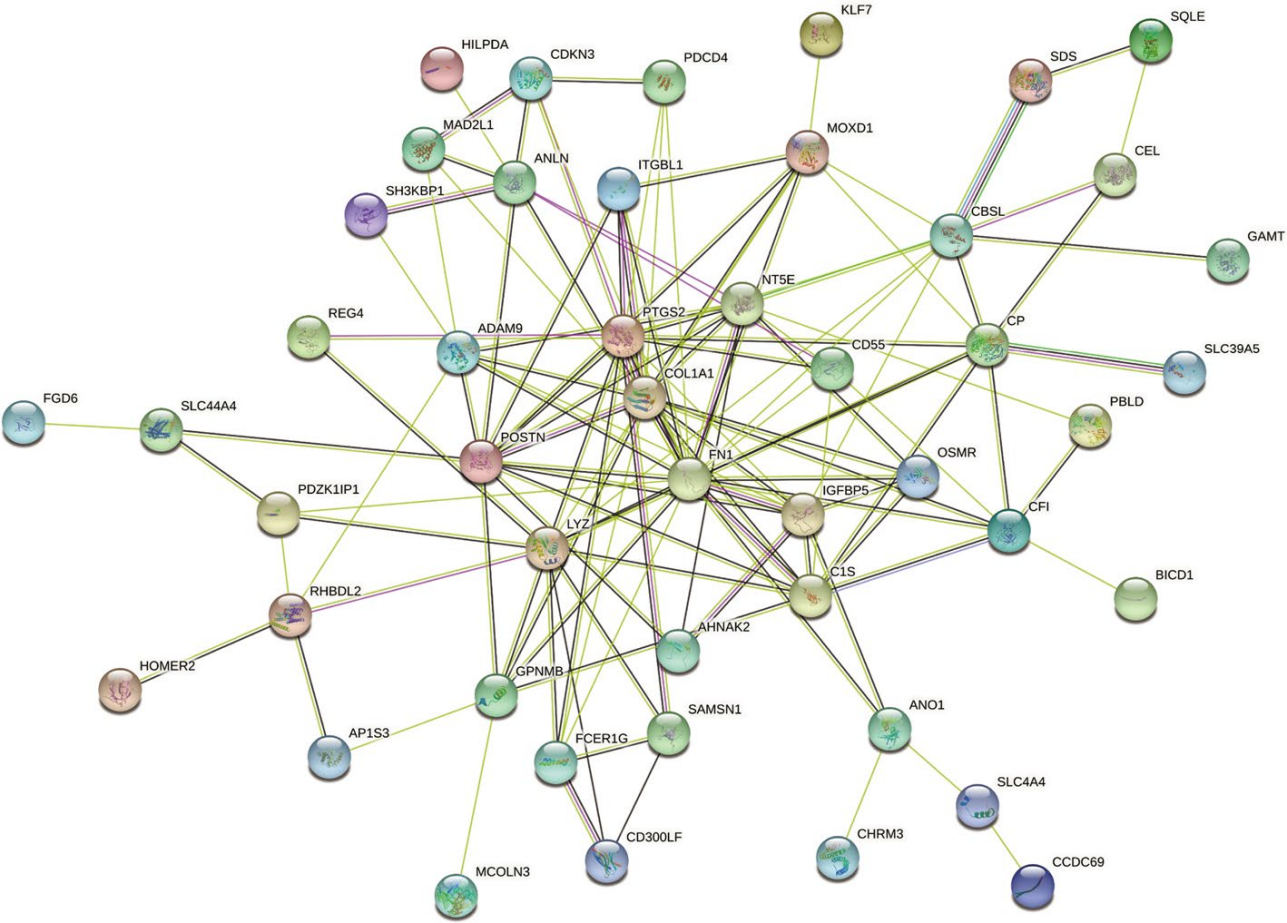
The protein–protein interaction (PPI) network was constructed to demonstrate the potential properties of DEGs

Firstly, Metascape analysis was carried out to find out the pathway and function of DEGs enrichment, and it was displayed by a bar diagram (Fig. 3A) and network diagram (Fig. 3B). Further, GO enrichment analysis was performed, and DEGs were mainly enriched in epidermis development (BP), endoplasmic reticulum lumen (CC), and sulfur compound binding (MF) (Fig. 4A, B). The KEGG pathway analysis revealed that Pancreatic secretion and Complement and coagulation cascades as important enrichment pathways for DEGs (Fig. 4C, D). At last, we protracted a PPI network diagram to explore the potential features of these DEGs (Fig. 5).

### Screening and verifying the feature genes of pancreatic cancer

**Fig. 6:**
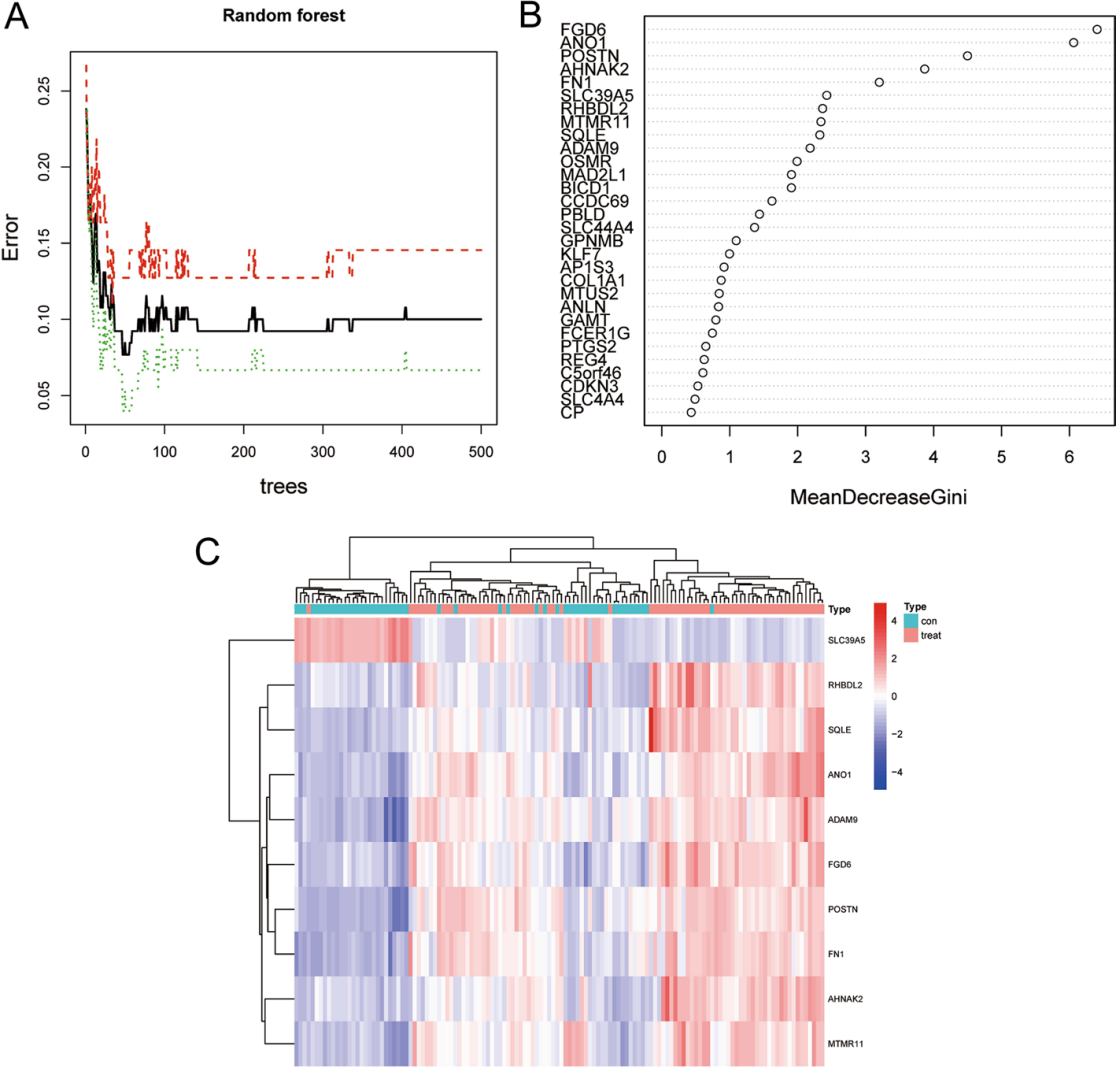
Filter out 10 characteristic genes. **A** According to the minimum point of cross-validation error, random forest tree filter out characteristic genes; **B** According to the importance score > 2, 10 characteristic genes were screened; **C** The heat map showed the feature genes filtered by the control group and the treat group

The random tree diagram showed the errors of the control group, the treat group, and all samples (Fig. 6A). We found the genes represented by the points with the smallest cross-validation errors and score these genes. The higher the score, the more important it is. Ten genes were selected according to the principle that the important score was > 2, namely FGD6, ANO1, POSTN, AHNAK2, FN1, SLC39A5、RHBDL2、MTMR11、SQLE, and ADAM9 (Fig. 6B). The heatmap presented the different expressions of the 10 feature genes in both groups (Fig. 6C).

### Neural network model construction and identification

**Fig. 7:**
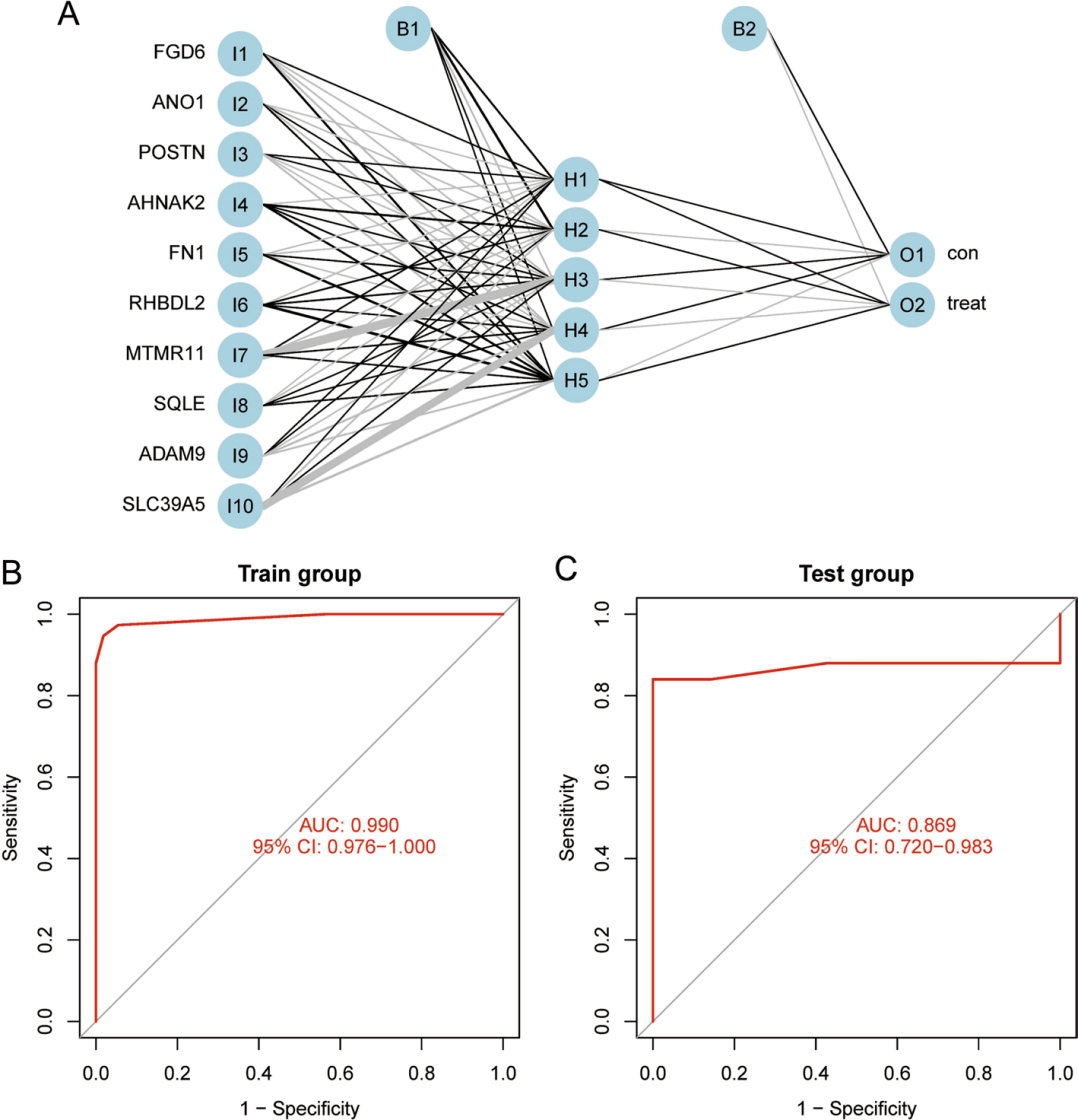
Construction of the neural network model. **A** Neural network model were built to predict genetic properties and consist of an input layer, hidden layer, and output layer; **B** Plotting ROC curve to detect the accuracy of the train group neural network model, the AUC was 0.990 (95% CI: 0.976–1.000); **C** ROC curve detection test group neural network model accuracy, the AUC was 0.869 (95% CI: 0.720–0.983)

According to gene scores and weights, a neural network model was constructed to identify sample attributes (Fig. 7A). The input layer was 10 genes with scores > 2, and 52/55 in the control group were correctly predicted, and 73/75 in the treat group were accurately predicted. Then, the ROC curves were established separately to detect the accuracy of the model in predicting the attributes of the sample. ROC curves of the control group and treat group were drawn to verify the accuracy of the model in predicting sample attributes. The area under the ROC curve of the train group is 0.990 (95% CI: 0.976–1.000) (Fig. 7B), which proved that the accuracy of its neural network model is high. Further external verification showed that the area under the test group curve is 0.869 (95% CI: 0.720–0.983) (Fig. 7C), which proved that the neural network model has high accuracy.

### Distribution of immune cells infiltrating

**Fig. 8:**
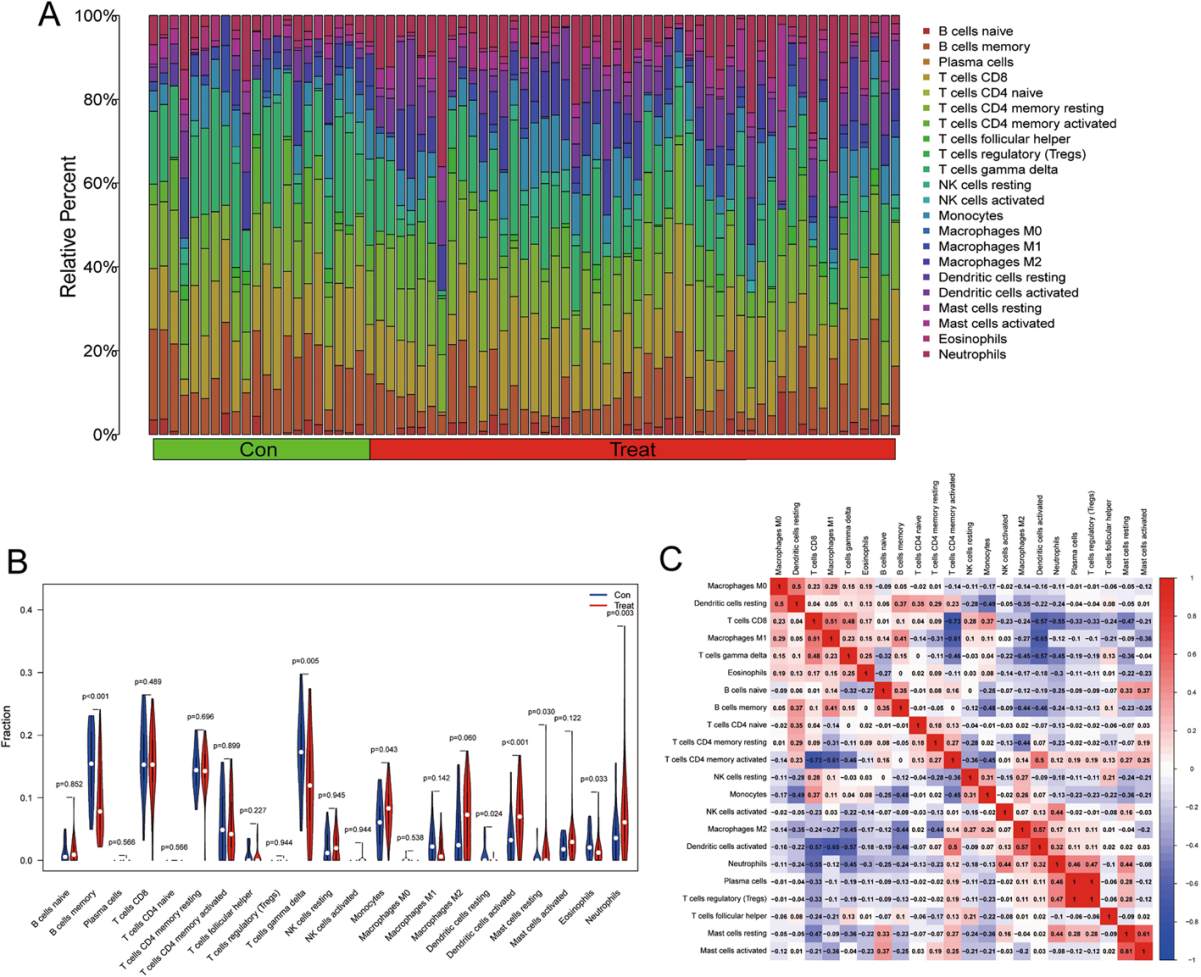
Infiltration immune cells of neural network model. **A** A histogram showed the distribution of infiltrating immune cells in the sample; **B** Different immune infiltrating cells differed significantly in control groups and treat groups, in the form of violins; **C** The relationships between immune cells were presented with heat

With the CiberSort algorithm, we calculated the scores of 22 kinds of immune cells in each sample to evaluate the immune infiltration state (Fig. 8A). The results showed that the activity of B cells memory and T cells gamma delta in the treat group declined significantly, while the activity of Neutrophils increased significantly (Fig. 8B). Finally, we drew a correlation heatmap to reveal the correlation between immune cells (Fig. 8C).

### The relationship between feature genes and prognosis

**Fig. 9:**
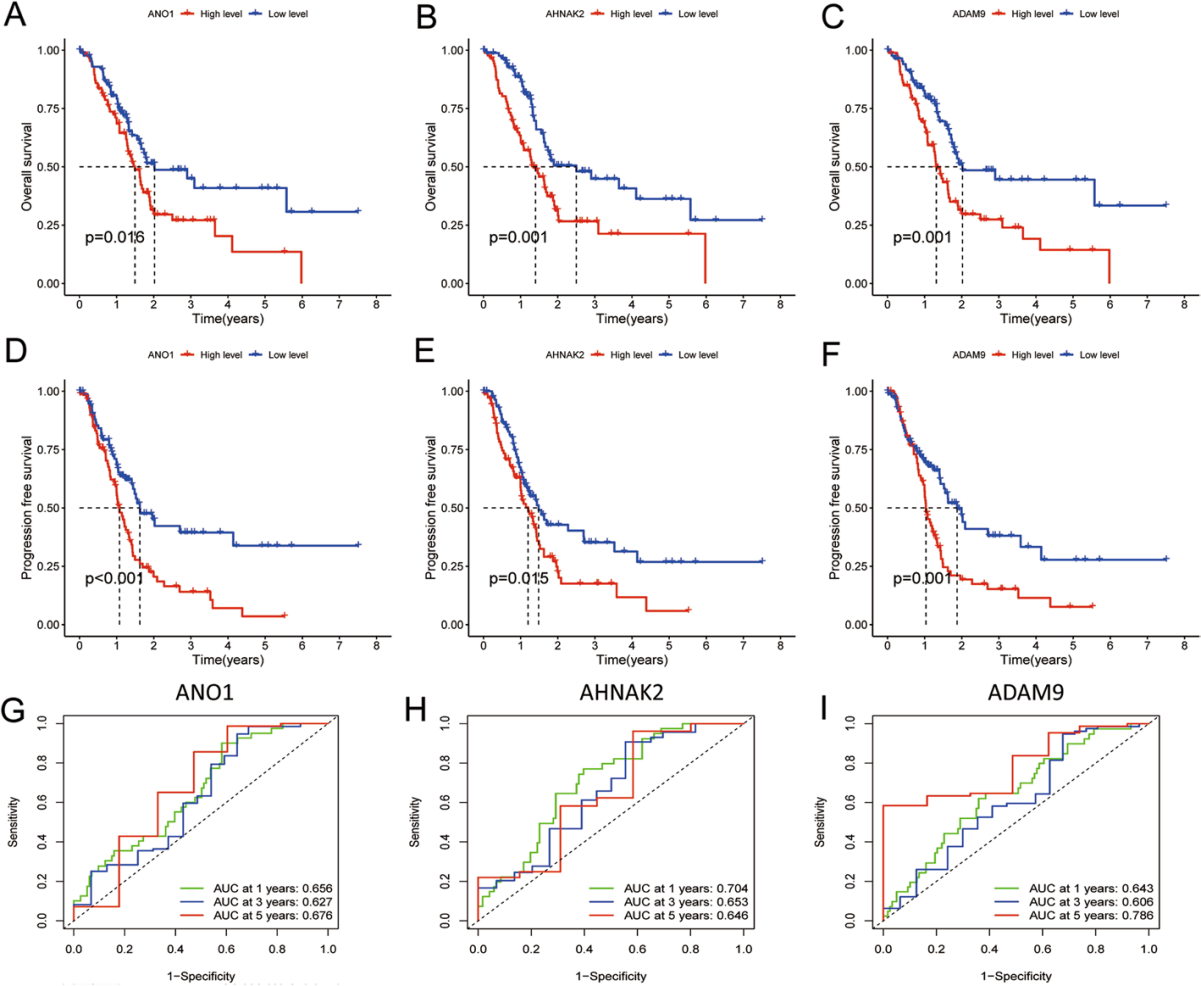
ANO1, AHNAK2 and ADAM9 were identified as characteristic genes for pancreatic cancer. **A**, **B**, **C** ANO1, AHNAK2 and ADAM9 predicted overall survival of pancreatic cancer respectively; **D**, **E**, **F** ANO1, AHNAK2 and ADAM9 predict the progression free survival of pancreatic cancer respectively; **G**, **H**, **I** ROC curves showed that ANO1, AHNAK2 and ADAM9 were effective in predicting the survival rate of pancreatic cancer patients at years 1, 3 and 5 years, and the area under the curve was > 0.60

In order to detect whether the feature genes in the model are closely related to the prognosis of pancreatic cancer, we obtained the gene expression profiles and corresponding survival information of 178 pancreatic cancer patient samples from TCGA and performed further OS, PFS, and ROC analysis. The results showed that only three feature genes—ANO1, AHNAK2, and ADAM9, were significantly associated with prognosis in all three analyses (*p* < 0.05) (Fig. 9 and Additional file 1: Fig. S1, S2 and S3), which means that these three feature genes may act as molecular markers for predicting the prognosis of pancreatic cancer patients.

### Immunohistochemical staining images validation

**Fig. 10:**
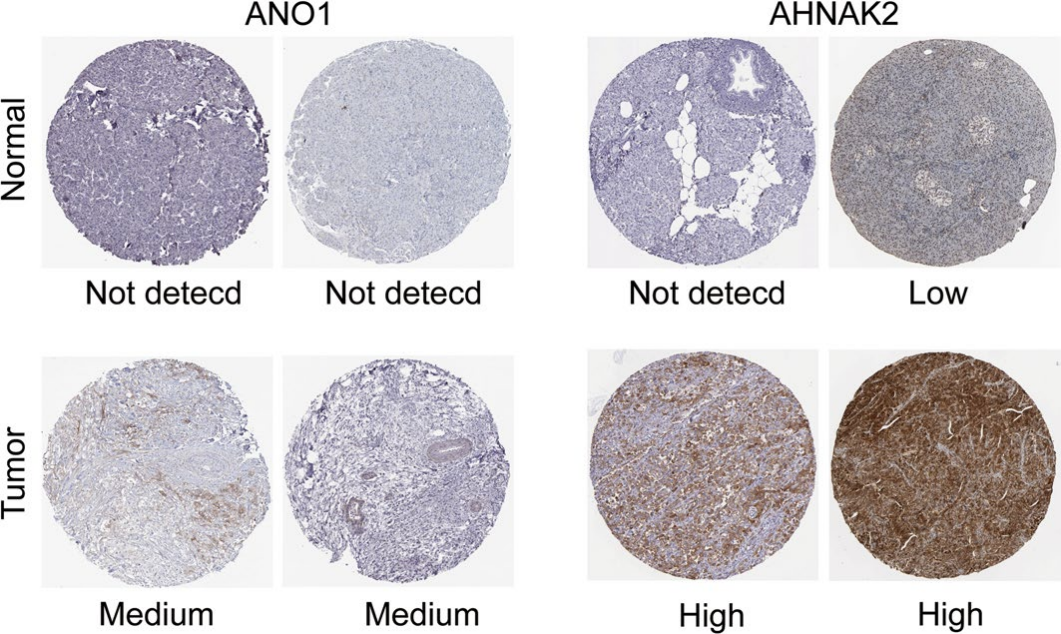
Immunohistochemical staining images of ANO1 and AHNAK2 were obtained from HPA (Human Protein Atlas), which showed high expression in tumor tissues

In the above analysis, we confirmed that all three feature genes were expressed higher in cancer tissue than in normal tissue at the transcriptome level. To further determine whether the feature genes are also present as proteins expressed in PDAC, we investigated the expression of these genes in HPA. This analysis could confirm the protein expression from feature genes utilizing data from IHC staining images. Except for ADAM9, which did not obtain images from HPA, immunohistochemical images of ANO1 and AHNAK2 showed high protein expression of genes in cancer tissue, especially AHNAK2 (Fig. 10). Although there were no ADAM9 images, previous literature have confirmed that ADAM9 was high protein expression in pancreatic cancer samples and promoted the development of pancreatic cancer [20, 21].

## Discussion

Since the 1960s, although the survival rates for some other cancers have improved dramatically, the survival rate of pancreatic cancer has remained relatively unchanged, and pancreatic cancer is still one of our deadliest malignant tumors. With the development of surgery, the surgical treatment of pancreatic cancer has made great progress. Nevertheless, the survival status and prognosis of pancreatic cancer have not been significantly improved. Globally, the 5-year survival rate of pancreatic cancer has been stagnant since the 1960s. In recent years, it has become the second largest cancer killer after lung cancer, not only with a short survival period but also with an extremely high mortality rate [22, 23]. Pancreatic cancer can occur in all years, especially above 60 years of the highest incidence. The onset of symptoms of pancreatic cancer is hidden and gradually progresses with time. The diagnosis of pancreatic cancer usually depends on symptoms. However, when the symptoms are obvious, the diagnosis is often late. Improving the survival rate largely depends on the early diagnosis of the disease. The common detection method contains endoscopic ultrasonography (EUS) or MRI/ magnetic resonance cholangiopancreatography. However, in high-risk groups, the accuracy of EUS/MRI screening is still unclear, and false positives or false negatives are frequent. For more accurate screening, which is both cost-effective and practical, simpler and more accurate screening methods, such as measuring biomarkers in blood samples, are needed.

In the course of our research, we first identified 55 DEGs and revealed the biological properties of these DEGs through GO and KEGG enrichment analysis. Then, 10 feature genes, namely FGD6, ANO1, POSTN, AHNAK2, FN1, SLC39A5, RHBDL2, MTMR11, SQLE, and ADAM9, were identified by random forest, and a neural network model was constructed with these 10 feature genes. ROC curve showed that the AUC of the train group and test group are 0.990 and 0.869, respectively, which indicated that the neural network model has high accuracy in predicting sample attributes.

Finally, according to the analysis of OS, PFS, and ROC curves, we further identified three important feature genes from 10 feature genes, namely ANO1 and AHNAK2, and ADAM9. ANO1 (TMEM16A) is a recently identified Ca(2 +)-activated Cl(−) channel (CaCC) that is upregulated in pancreatic ductal adenocarcinoma (PDAC) [24], it is also a gene up-regulated in pancreatic cancer samples. AHNAK2 is a member of the AHNAK2 family. Lu's past research has demonstrated that AHNAK2 is abundantly expressed in pancreatic tissue, and it is closely related to poor prognosis [25]. There have been many studies on ADAM9 and tumors, it has been found that circ-ADAM9 also showed an upward regulatory trend in pancreatic cancer. The stepwise mechanism shows that circ-ADAM9 can reduce the inhibitory effect of miR-217 on the oncogene PRSS3, thus activating ERK/VEGF signaling pathway. In vivo, circ-ADAM9 silencing or miR-217 overexpression delays the growth of the tumor, and their combination shows an obvious inhibitory effect on tumourigenicity [26]. Previous studies have shown that these genes play a role in different tumors, and we have analyzed these three genes through bioinformatics that may be associated with pancreatic cancer, but further experiments are needed to verify this.

The microenvironment of a tumor has a significant impact on its growth [27]. The host immune response represented by tumor infiltrating immune/inflammatory cells is one of the main participants in the tumor microenvironment, and tumor infiltrating immune/inflammatory cells will become a significant marker for assessing the features of the tumor immune microenvironment and immune monitoring of untreated patients and tumor tissues. B cells memory and T cells gamma delta play an important role in the human innate immune system. Studies have shown that B cells memory and T cells gamma delta have a significant anti-tumor effect in the process of cancer development. In addition, the study also showed that the increase of Neutrophil content in cancer patients indicates a bad prognosis. In this study, we found that the content of B cells memory and T cells gamma delta decreased, while the content of Neutrophils increased significantly in the treat group.

The Artificial neural network is based on machine learning to predict gene expression in pancreatic cancer. In cancer genomics, the neural network model is a promising tool to extract advanced features and learn prognosis information from multiple cancer data sets [28]. The random tree is a method used in many fields because it can be applied to complex data, and small sample data, deal with complex relationships and correlations, and predict confounding factors, which provides great convenience for bioinformatics [29].

Our experimental data are all from the GEO database, which is a single database source, which may limit the range of the final output feature genes to some extent. Considering different tumor classifications and more subtypes, it would be more meaningful to predict different types of pancreatic cancer more accurately. In addition, cell experiments and animal experiments are necessary for further confirmation.

## Conclusion

Based on the GEO database, we constructed a neural network model containing 10 feature genes, and finally screened out 3 feature genes most closely related to pancreatic cancer. This is important for predicting pancreatic cancer and assessing prognosis.

## Supplementary Information

**Additional file 1.** Overall survical (OS) analysis, progression free survival (PFS) analysis and ROC curves of 7 feature genes in pancreatic cancer patients.

## Data Availability

The datasets generated and/or analysed during the current study are available in the GEO database repository, https://www.ncbi.nlm.nih.gov/geo/ to datasets.
