# Supplementary material for: A neural network model to screen feature genes for pancreatic cancer
Source: BMC Bioinformatics. 2023 May 11;24:193. doi: 10.1186/s12859-023-05322-z (PMC10176951; doi:10.1186/s12859-023-05322-z)
Supplement: Supplementary file 1 — Additional file 1. Overall survical (OS) analysis, progression free survival (PFS) analysis and ROC curves of 7 feature genes in pancreatic cancer patients. [file 12859_2023_5322_MOESM1_ESM.doc]

A B C


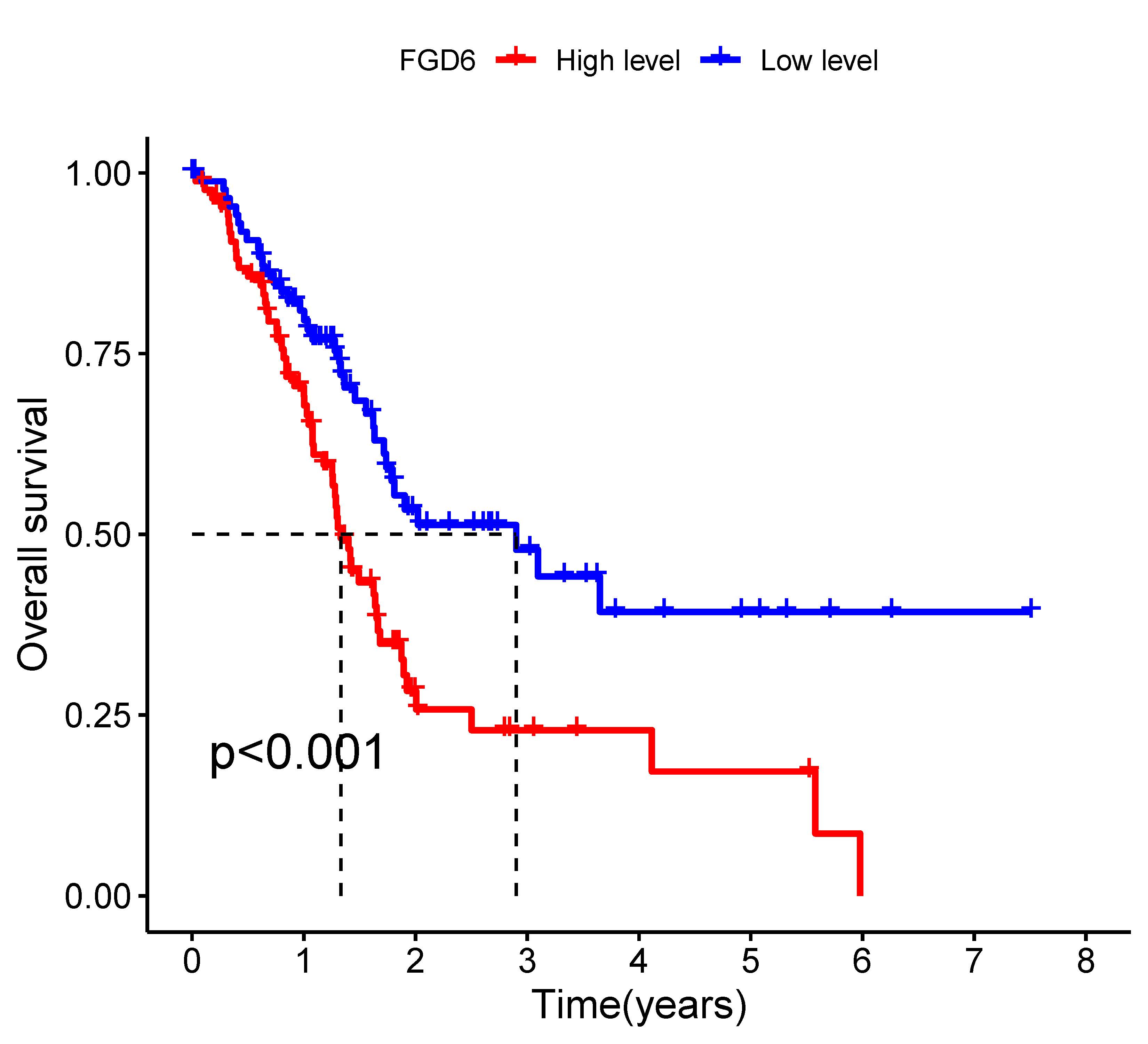

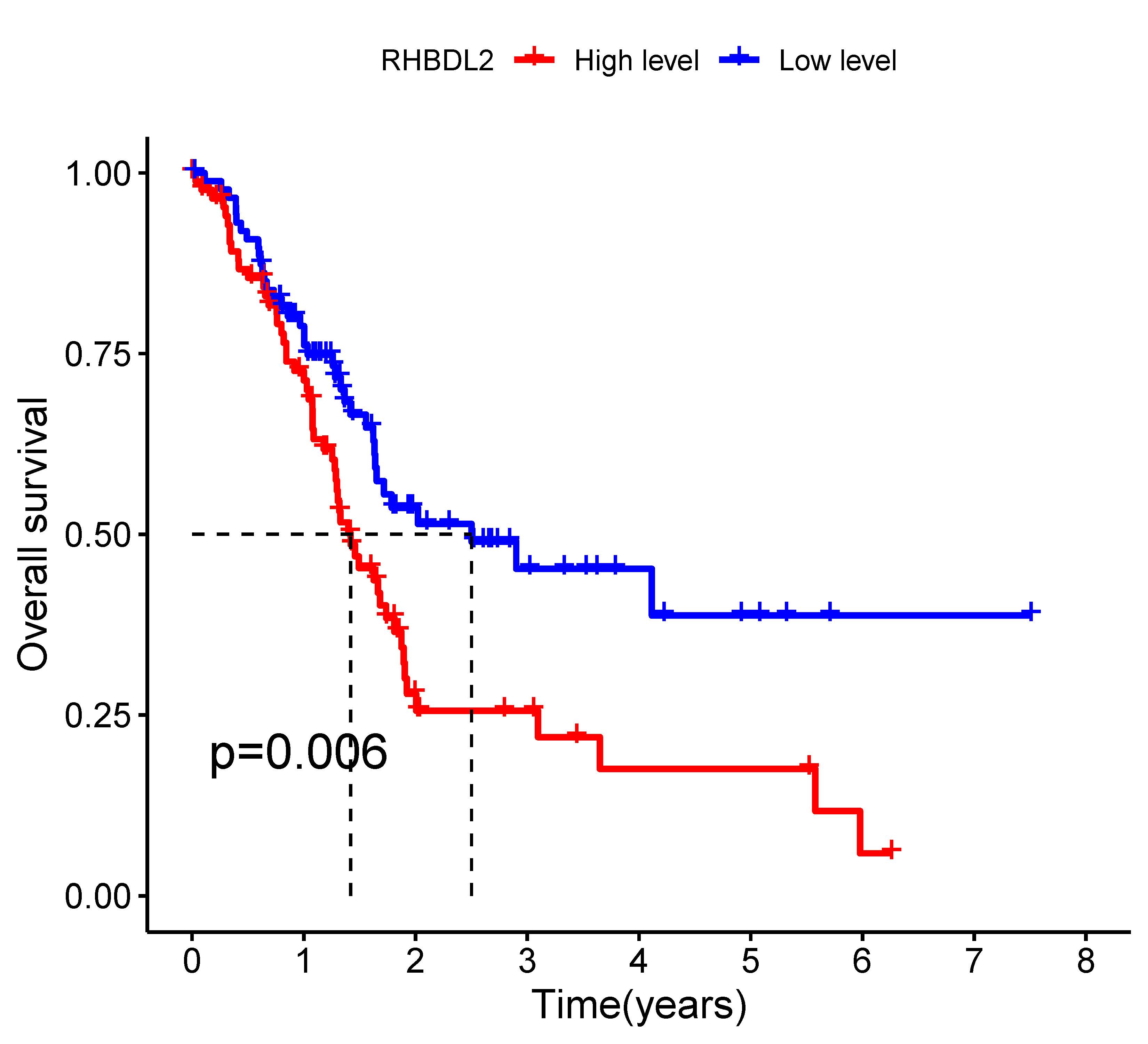

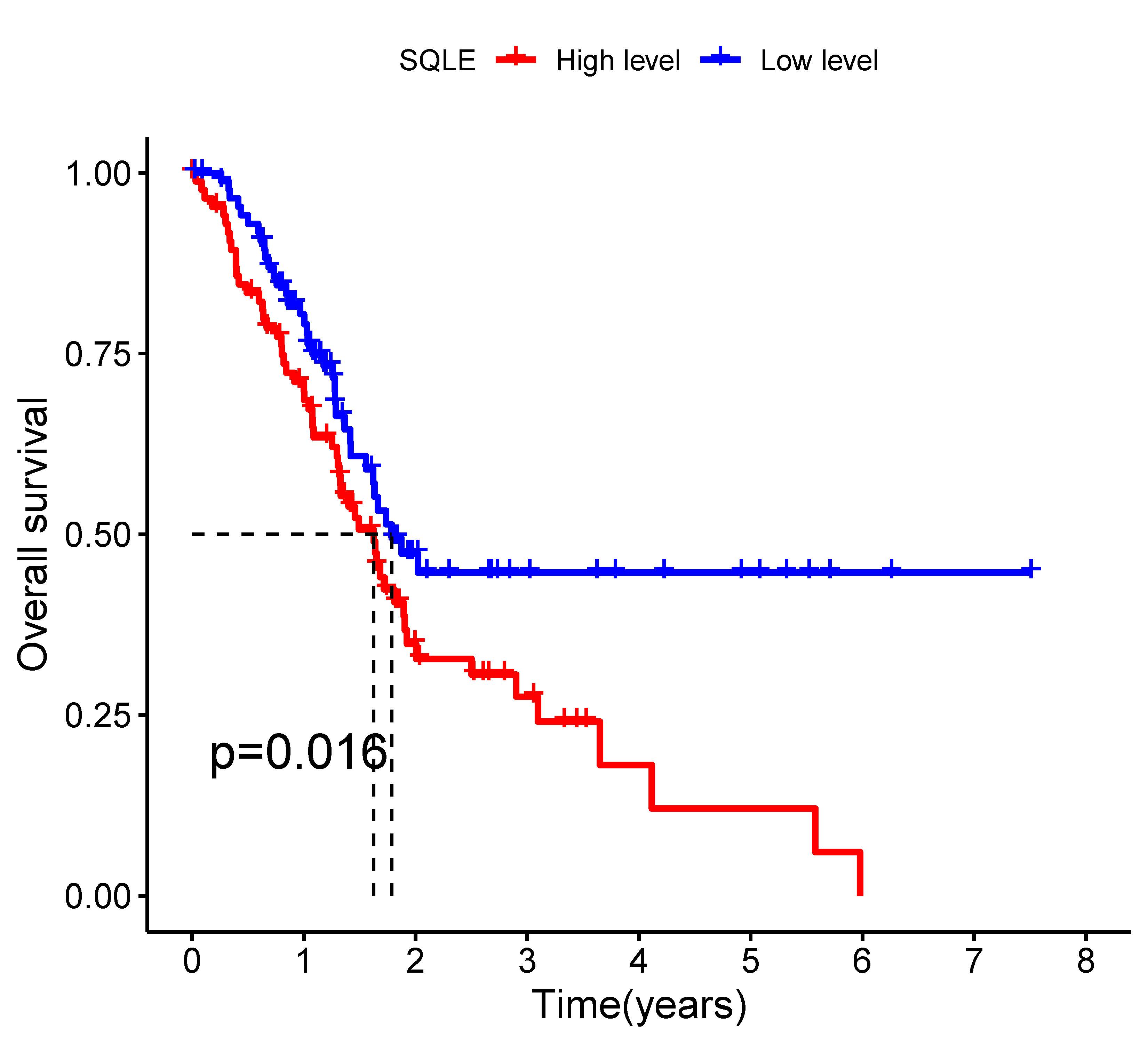


D E F


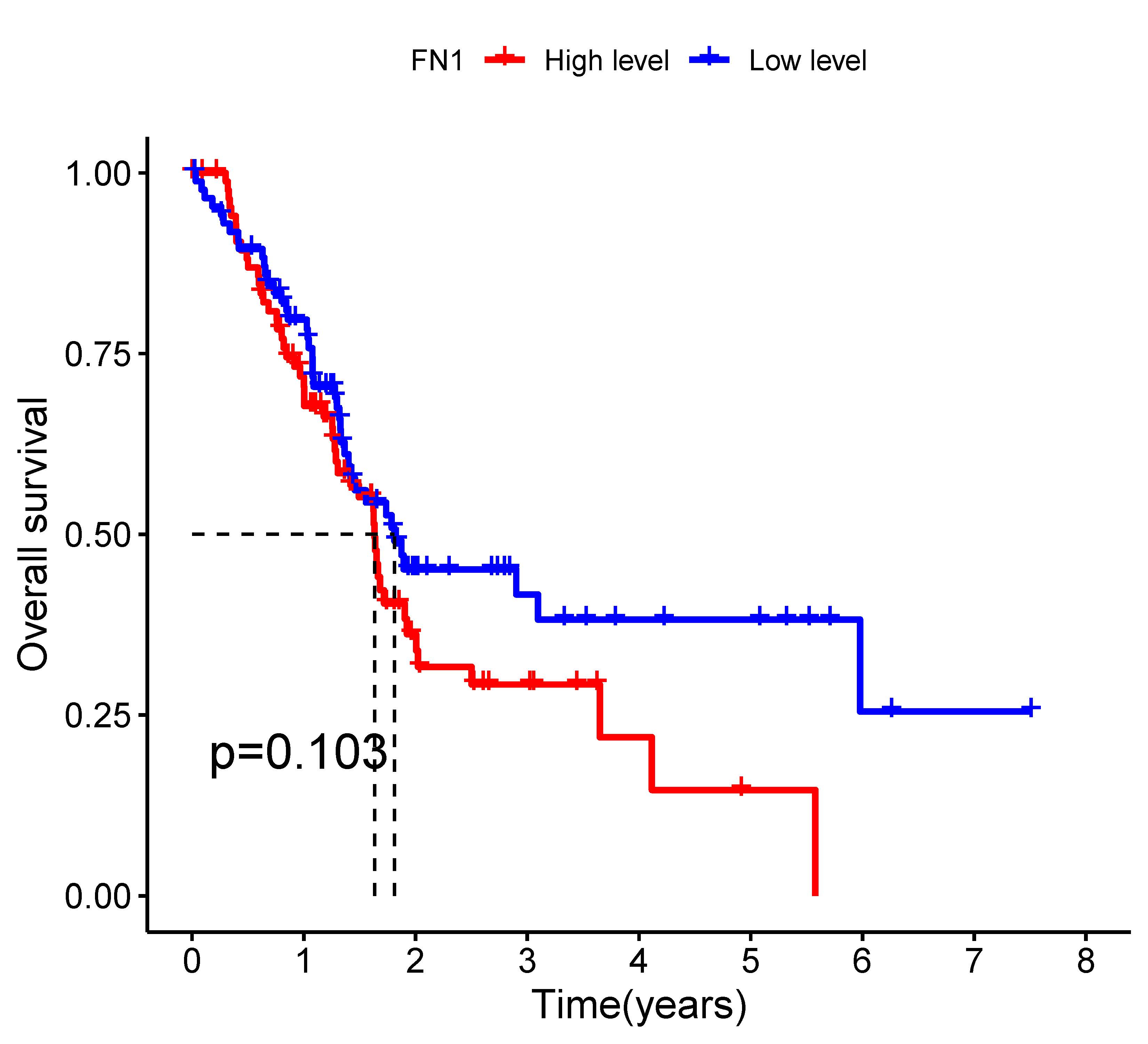

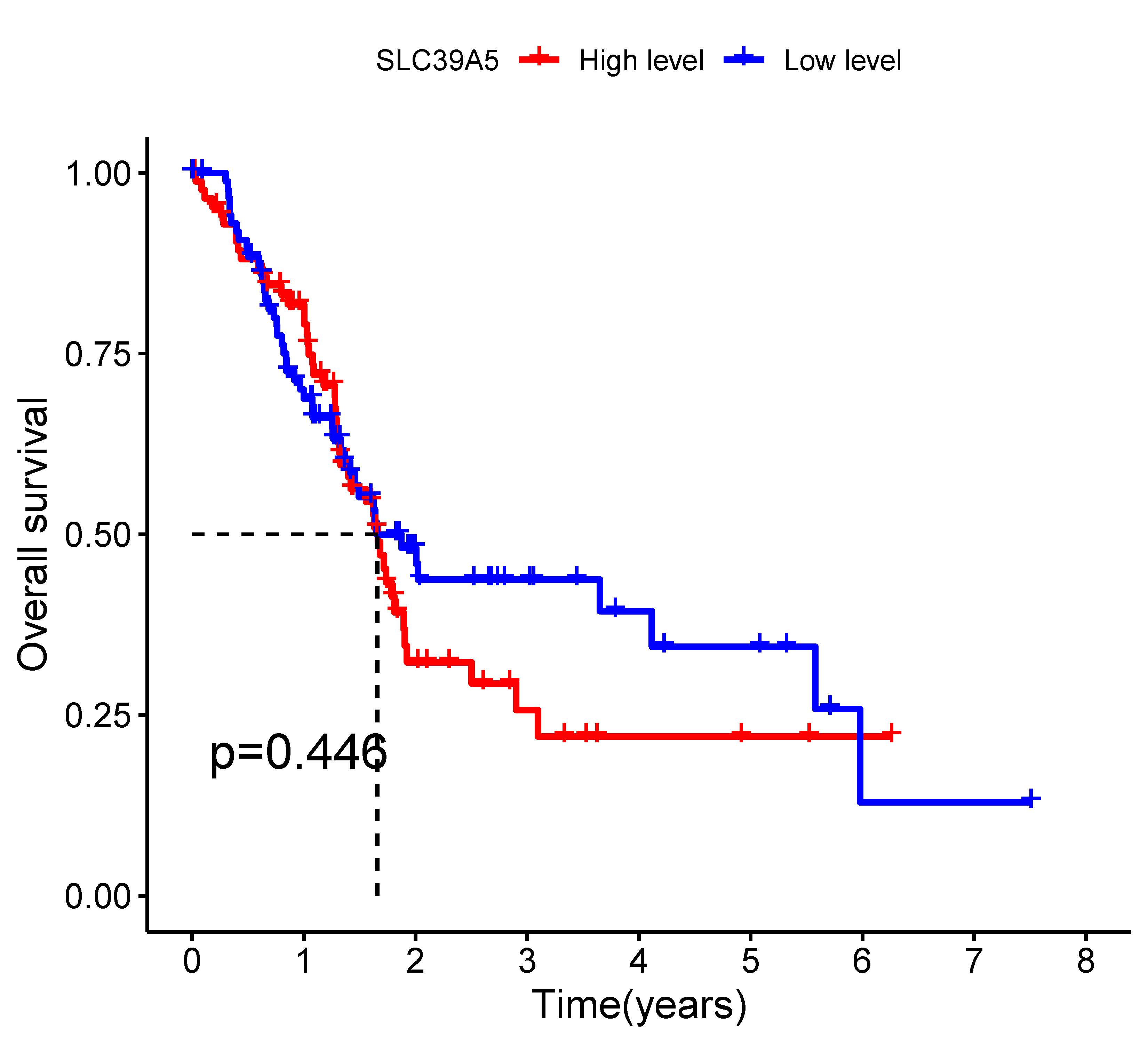

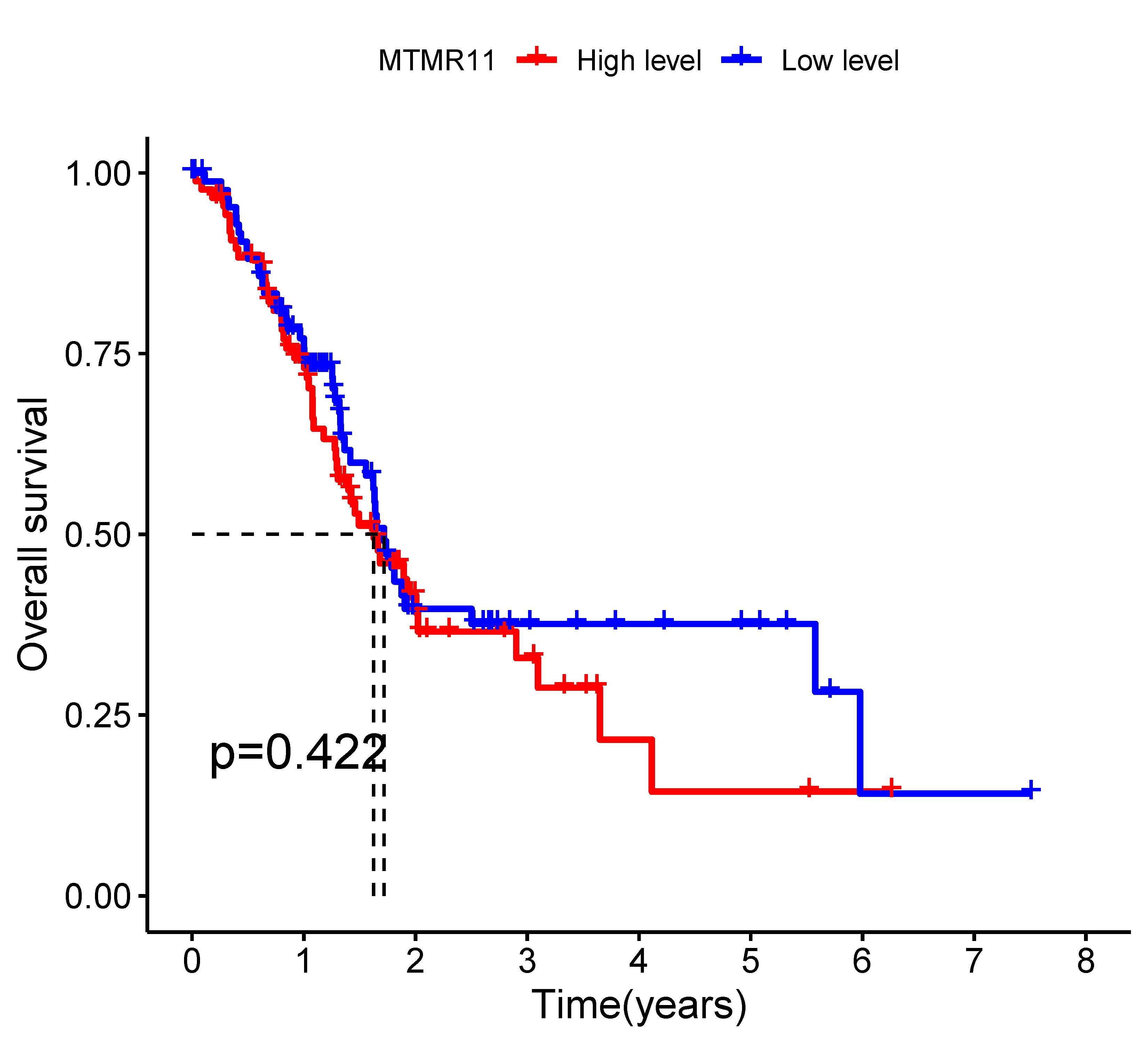


G


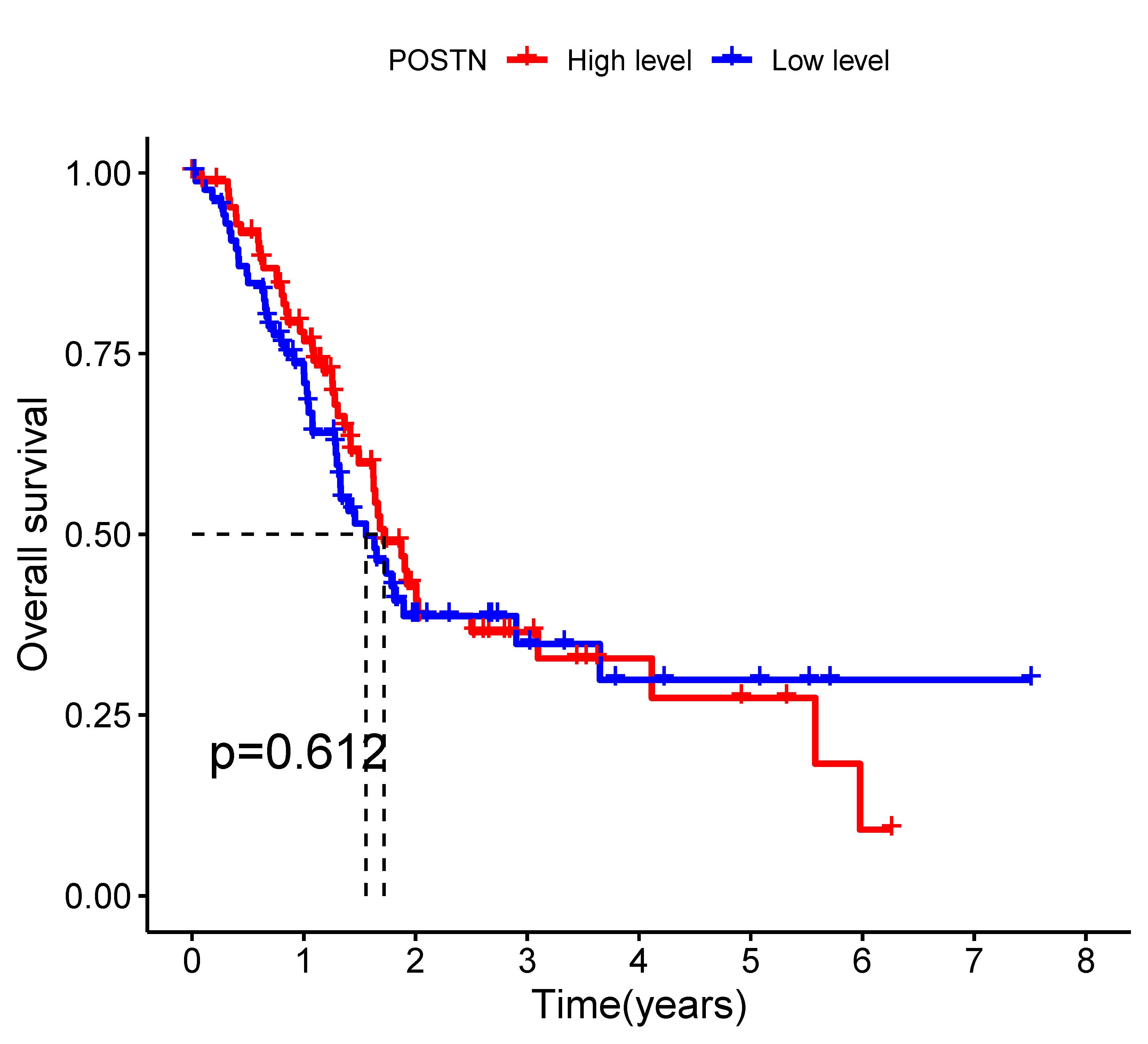


Supplementary fig.1

Overall survical (OS) analysis of 7 feature genes in pancreatic cancer patients by KM plotter. **A** FGD6 **B** RHBDL2 **C** SQLE **D** FN1 **E** SLC39A5 **F** MTMR11 **G** POSTN

A B C


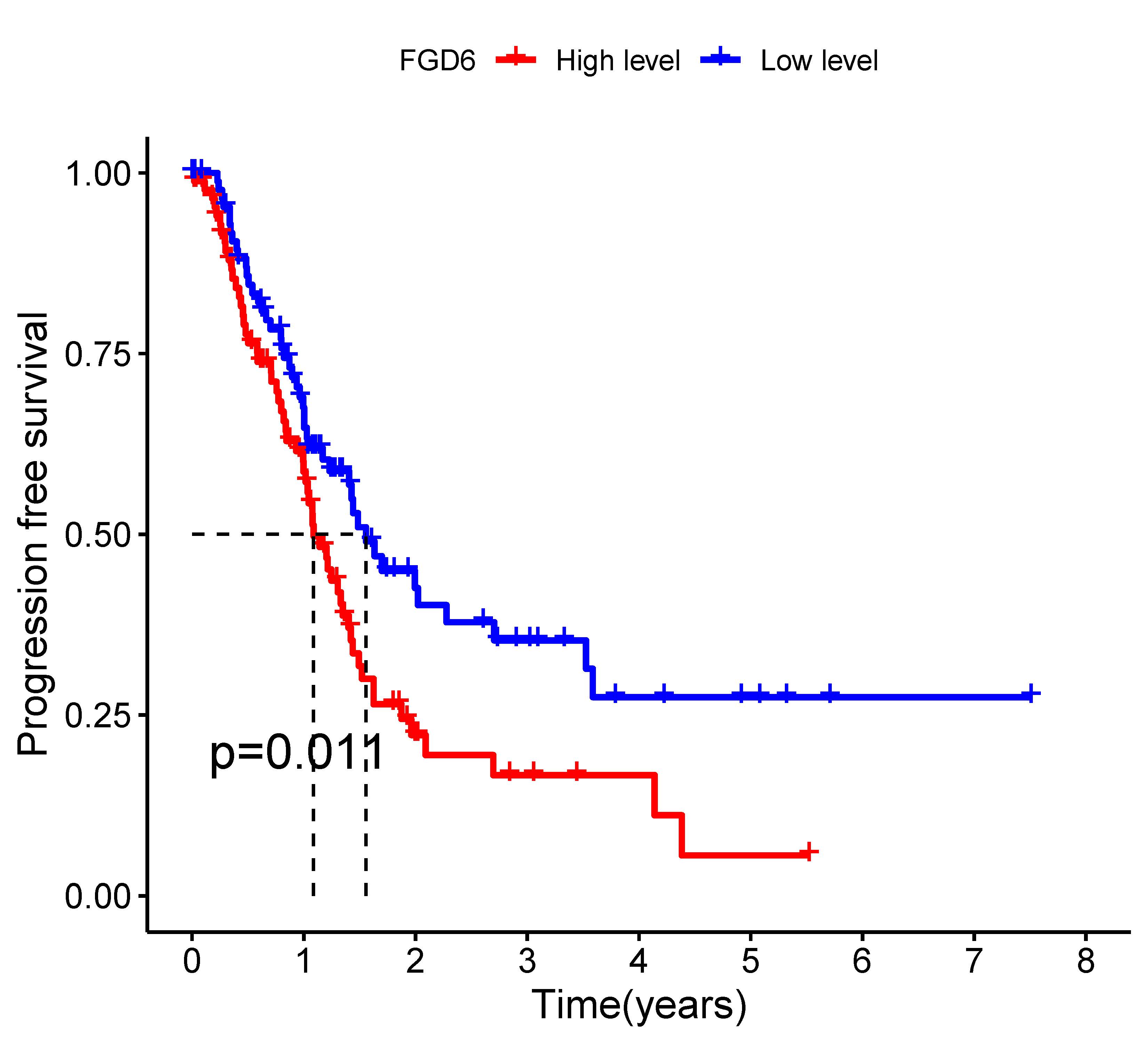

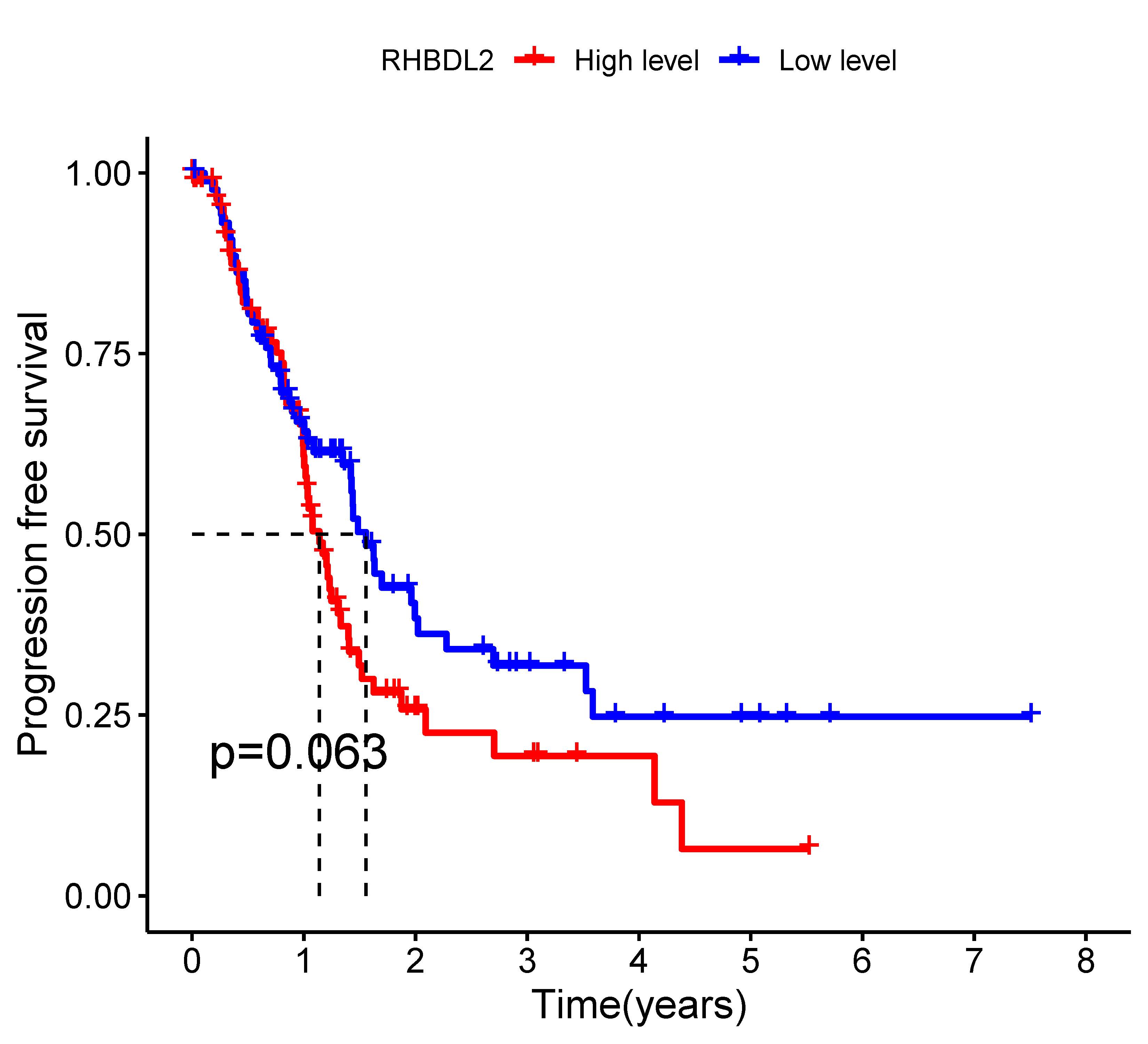

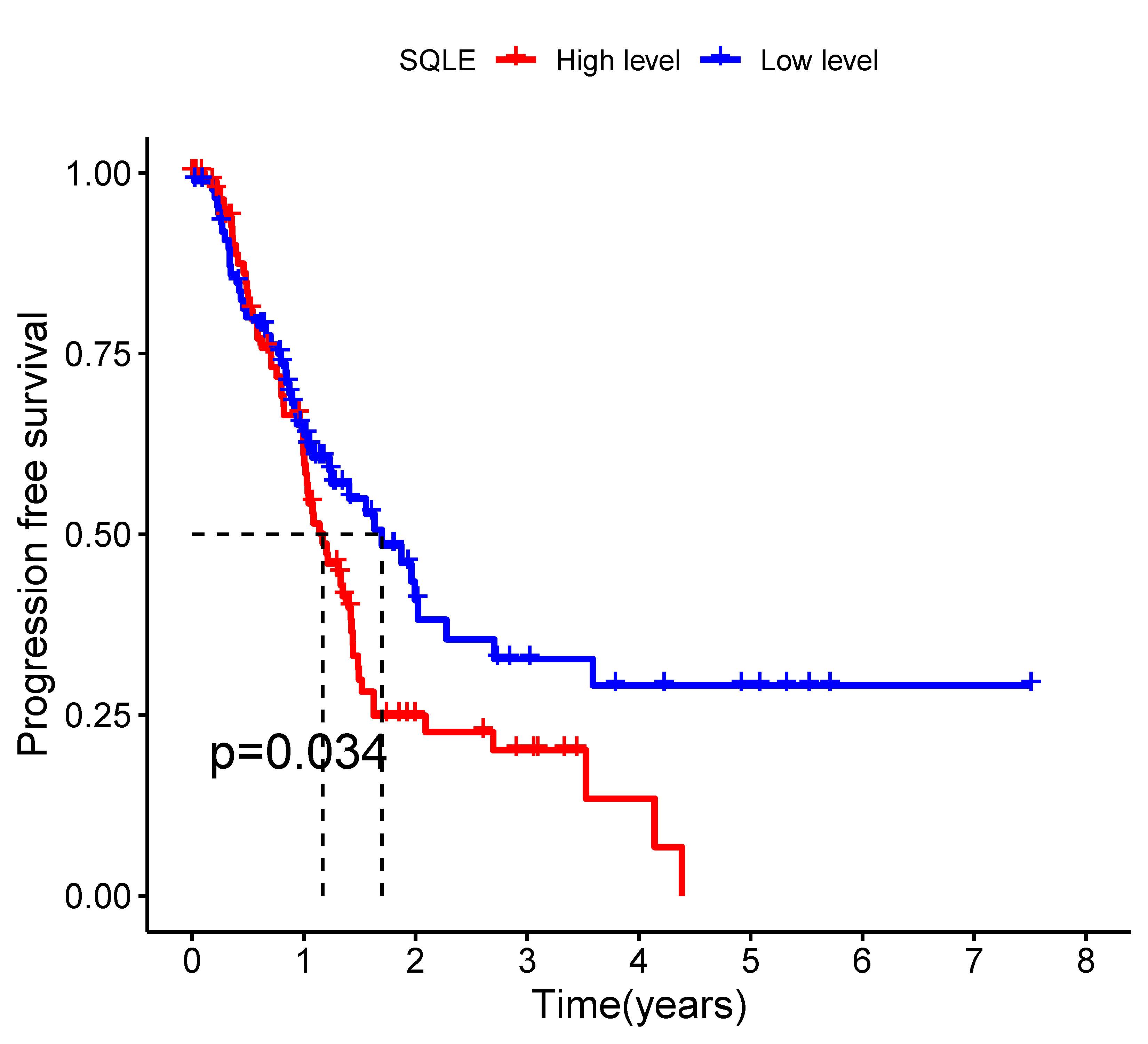


D E F


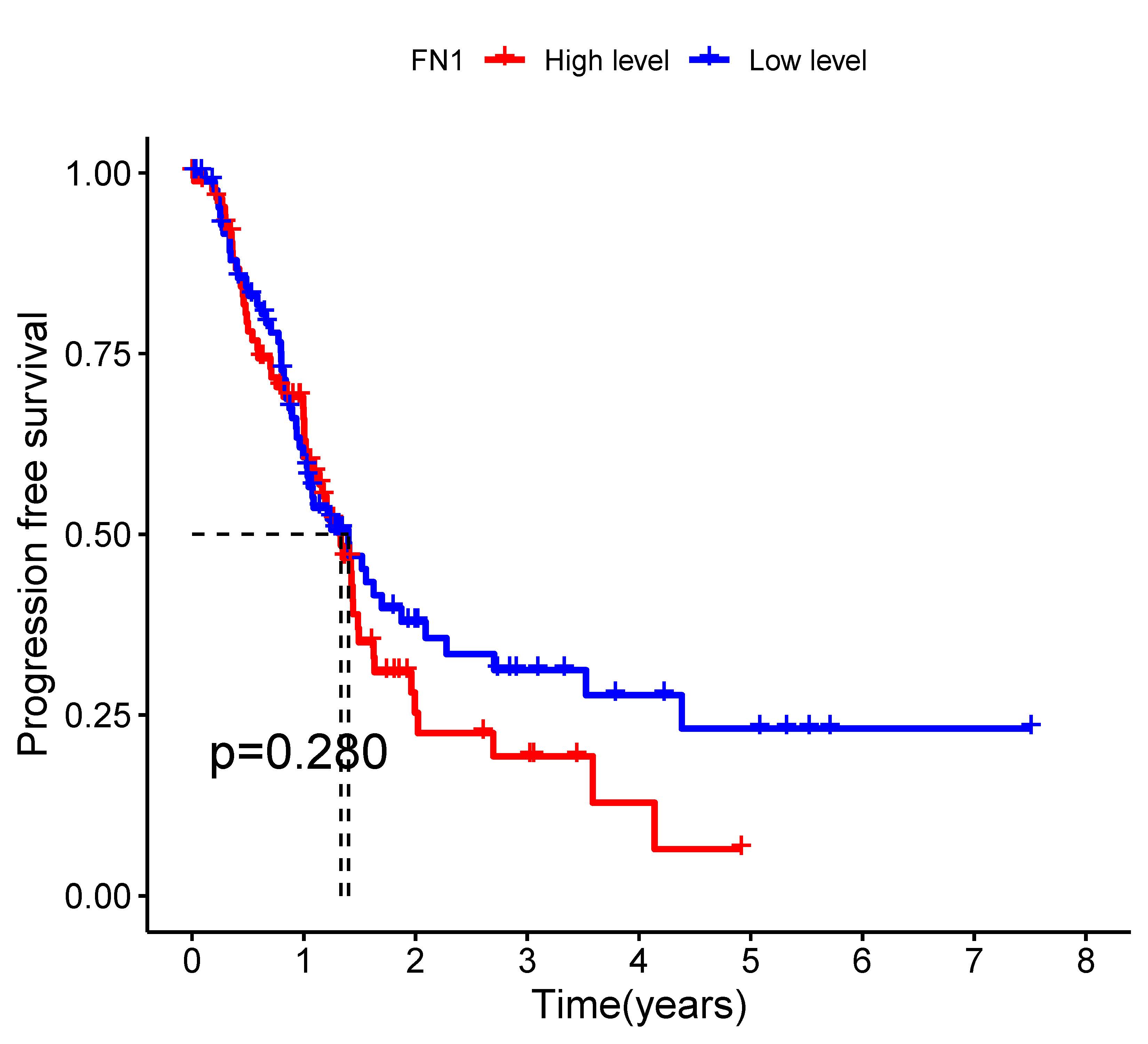

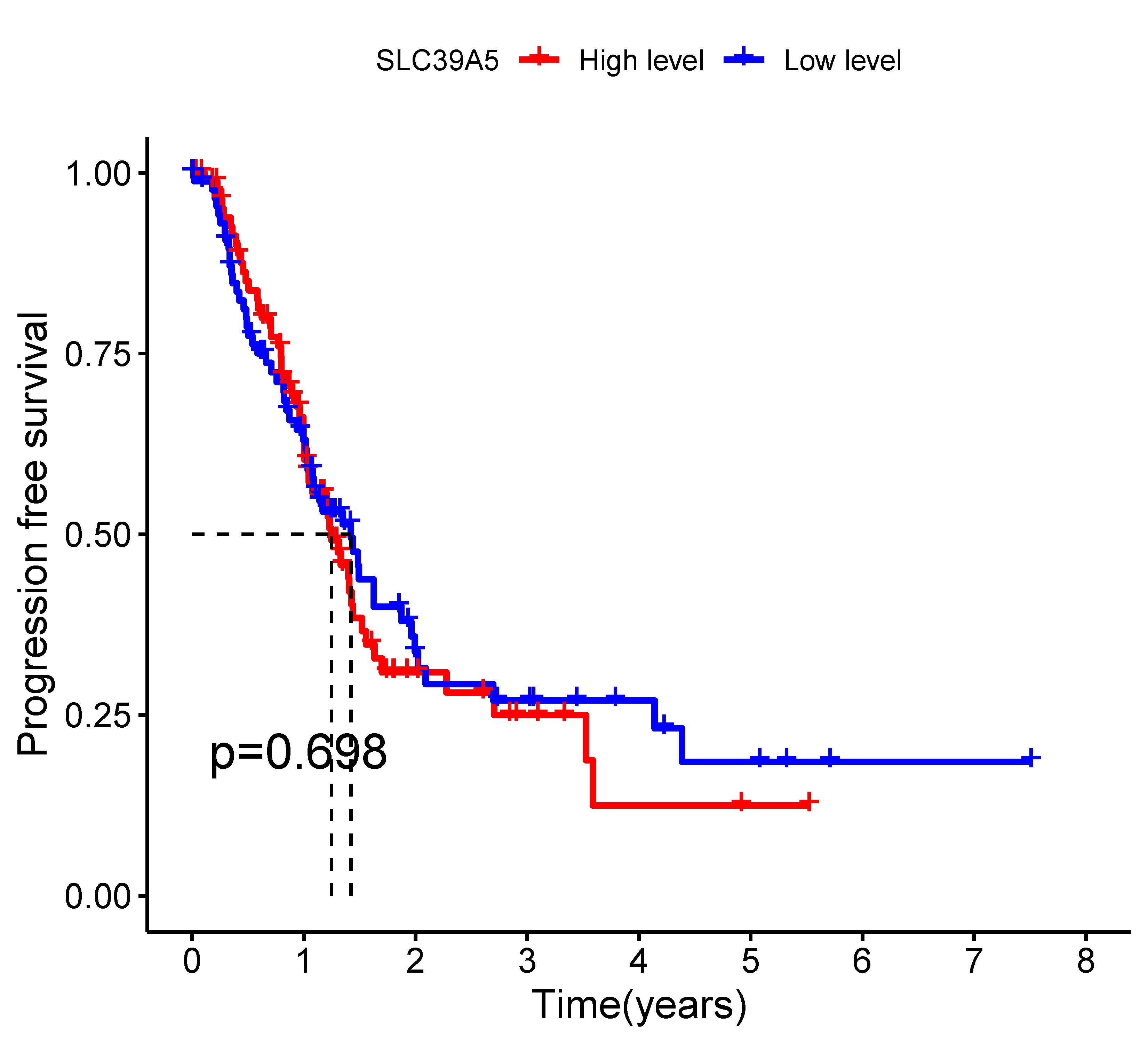

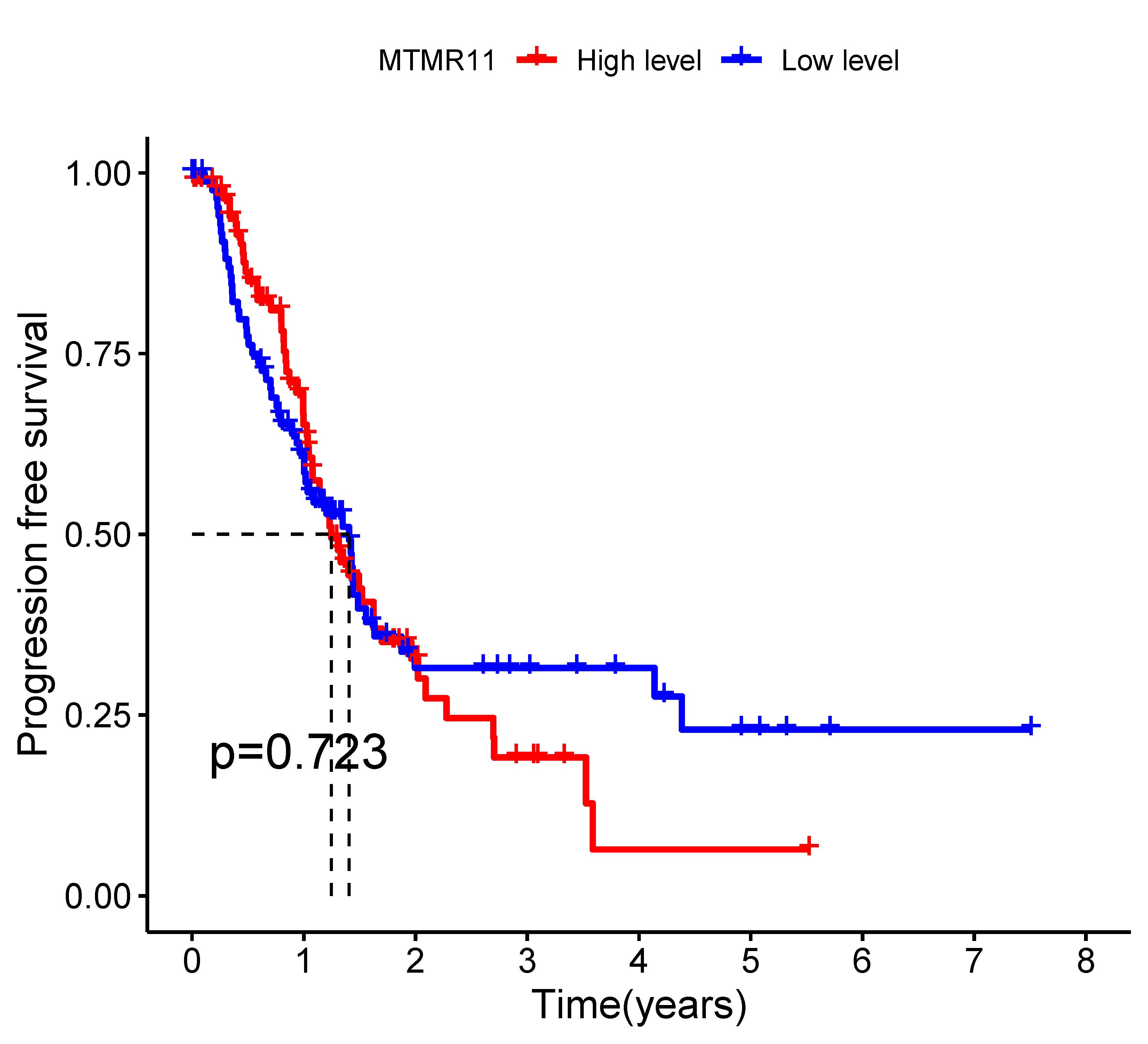


G


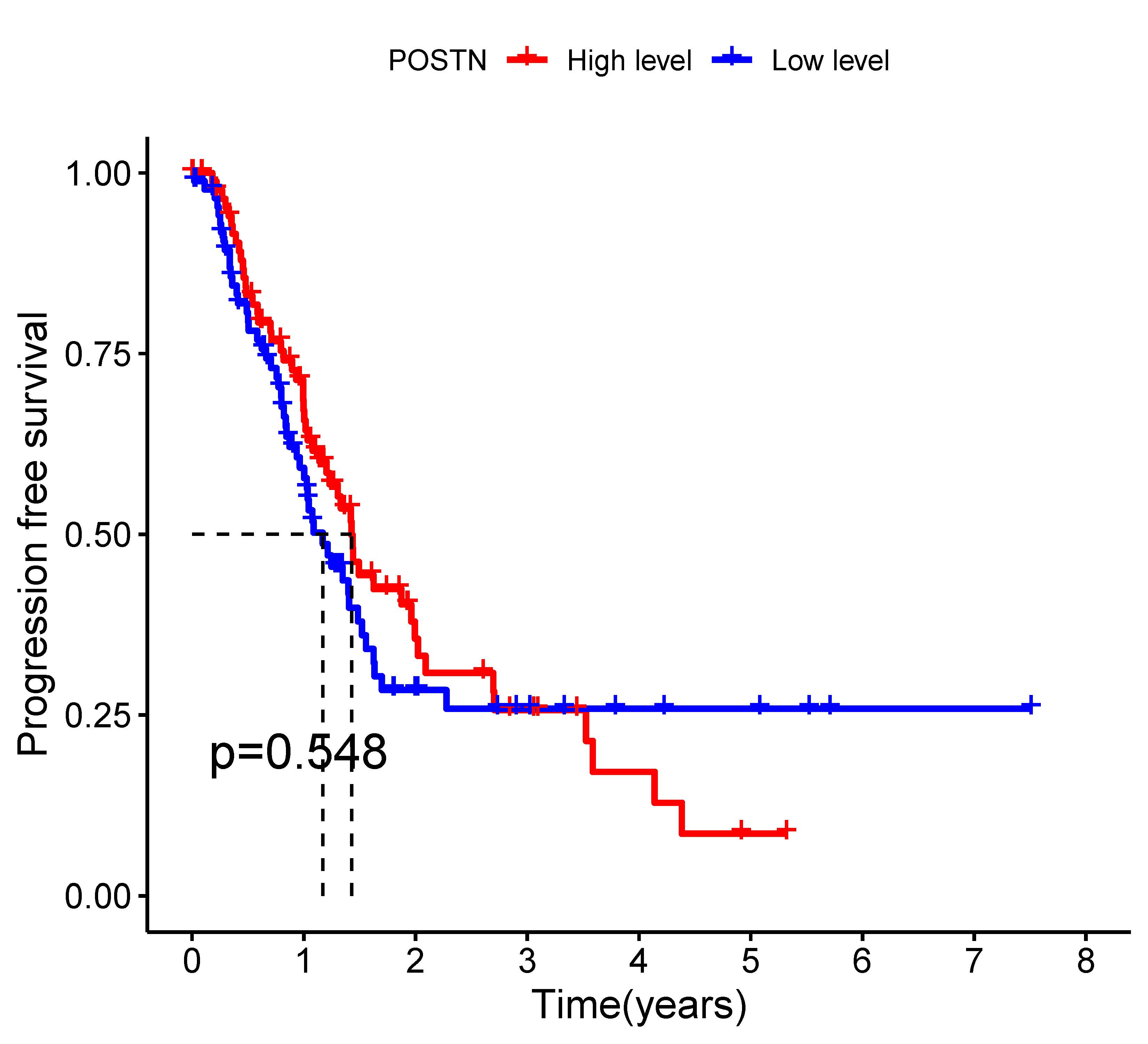


Supplementary fig.2

Progression free survival (PFS) analysis of 7 feature genes in pancreatic cancer patients by KM plotter.

**A** FGD6 **B** RHBDL2 **C** SQLE **D** FN1 **E** SLC39A5 **F** MTMR11 **G** POSTN

A B C


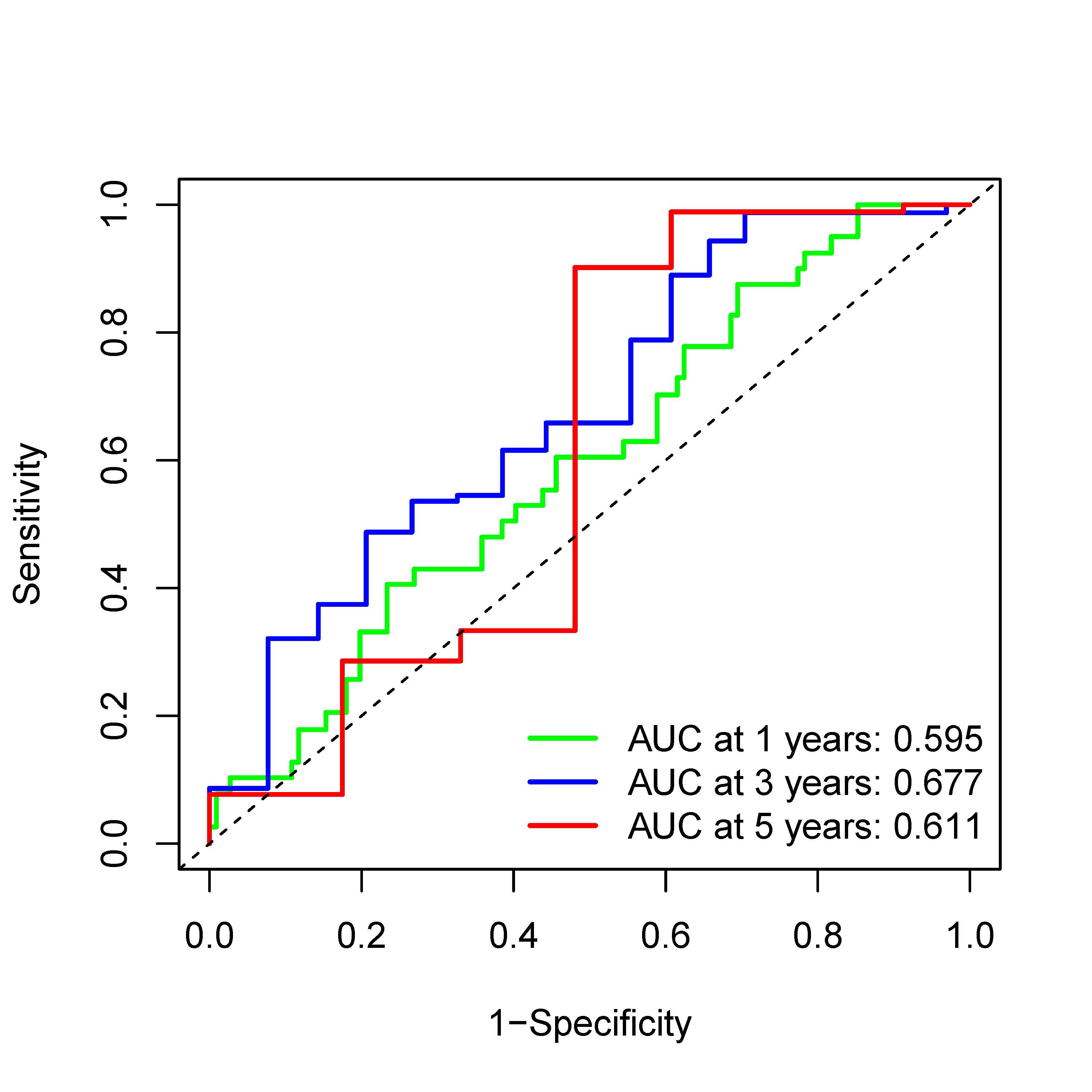

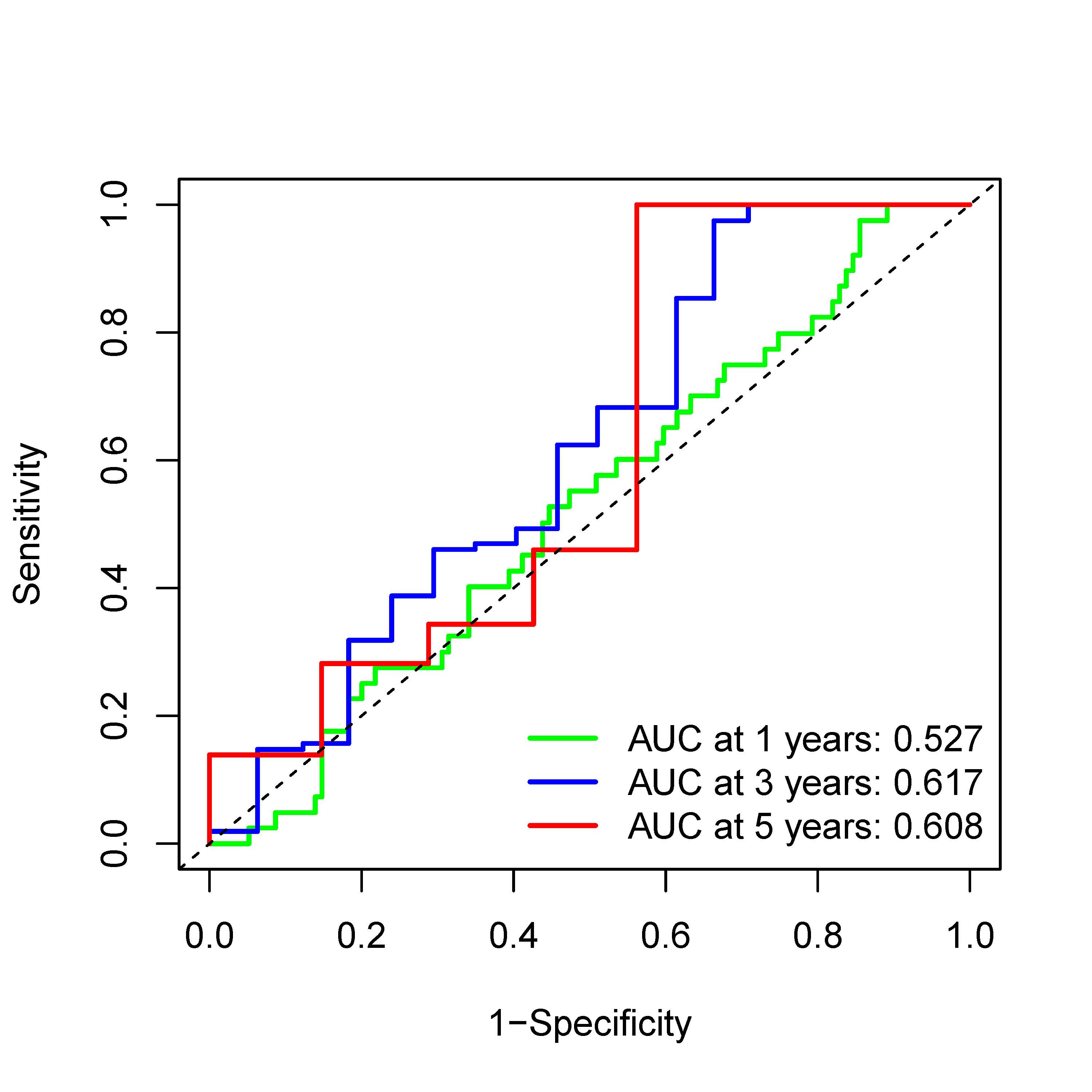

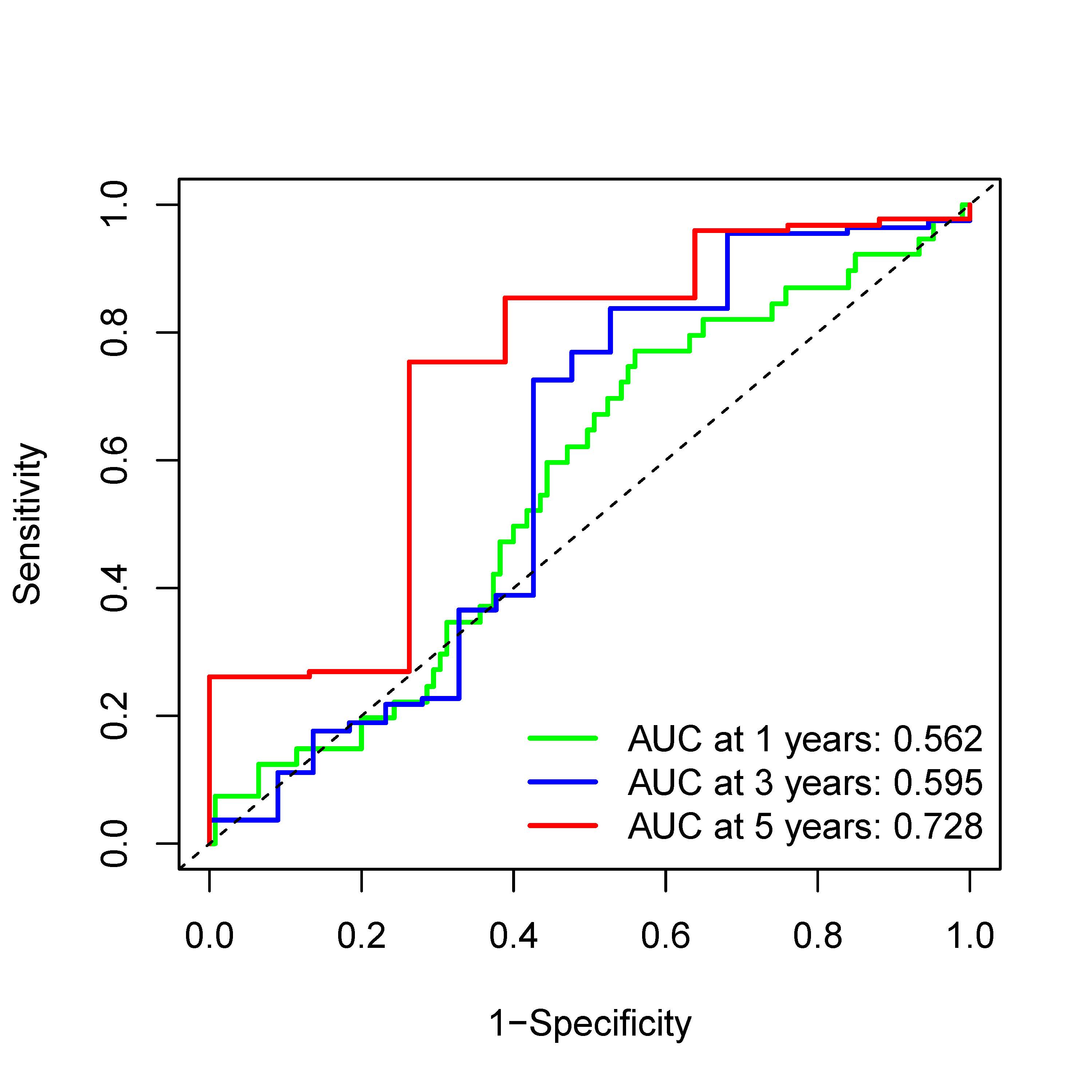


D E F


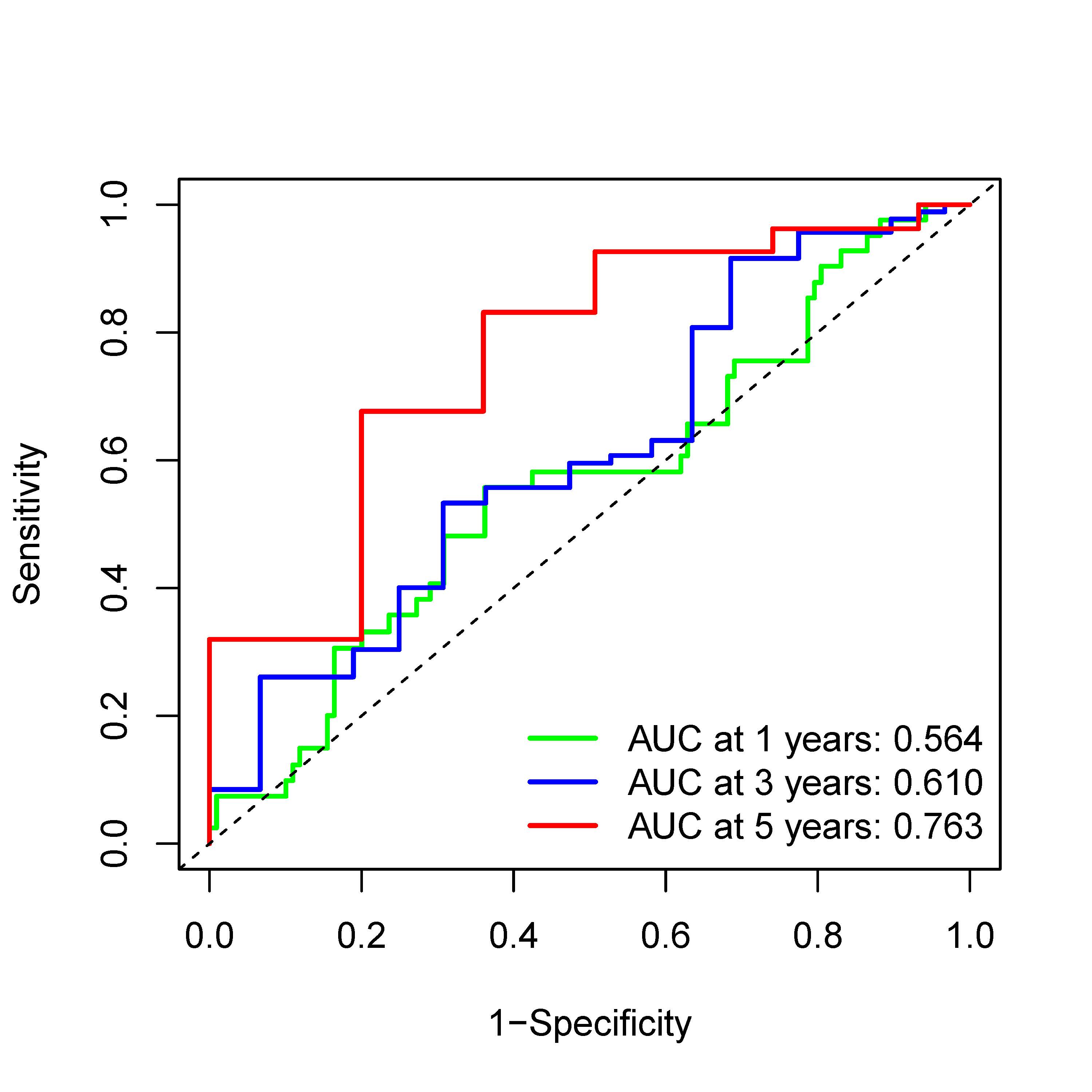

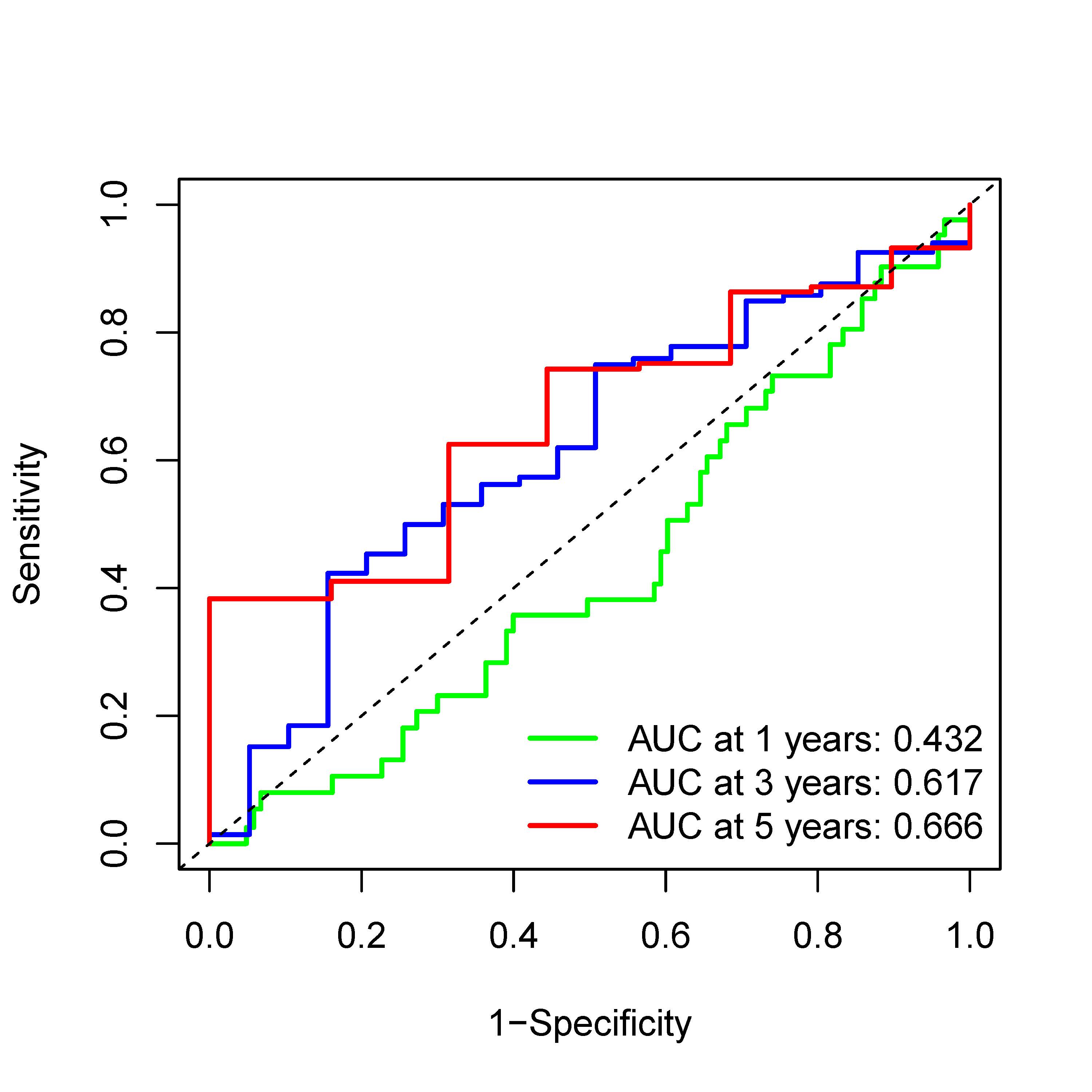

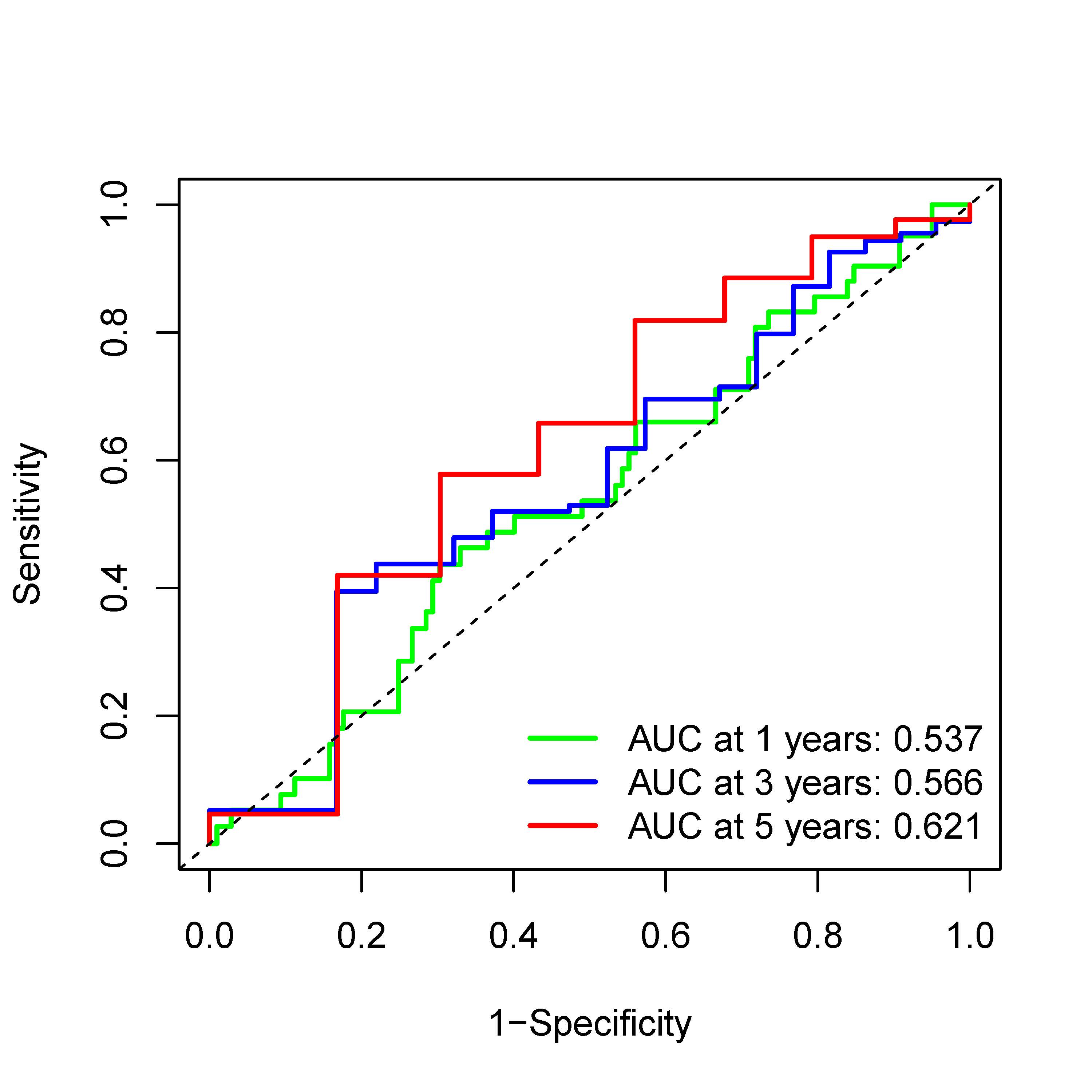


G


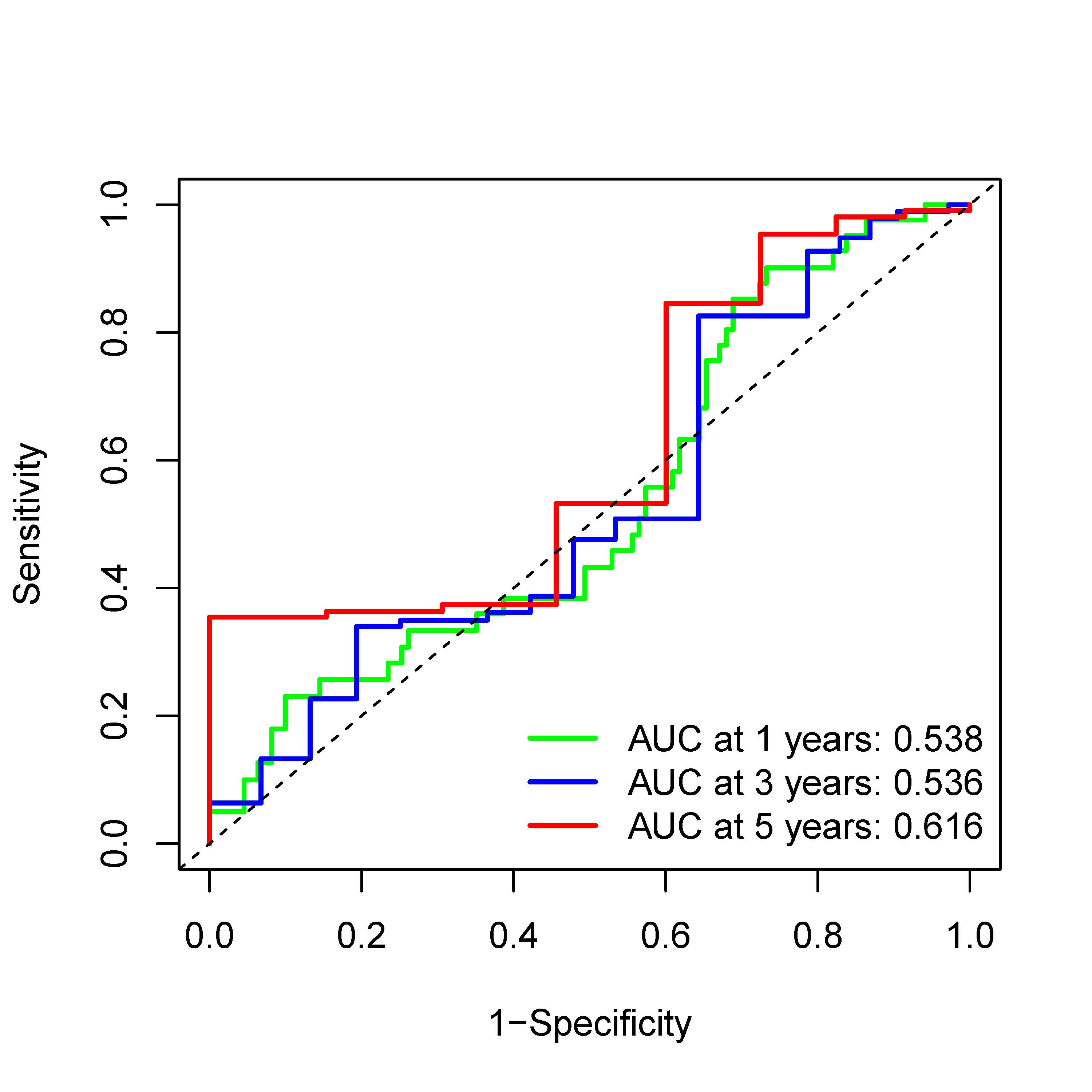


Supplementary fig.3

ROC curves of 7 feature genes in pancreatic cancer patients by KM plotter.

**A** FGD6 **B** RHBDL2 **C** SQLE **D** FN1 **E** SLC39A5 **F** MTMR11 **G** POSTN
